# Nonlinear Actomyosin Elasticity in Muscle?

**DOI:** 10.1016/j.bpj.2018.12.004

**Published:** 2018-12-13

**Authors:** Alf Månsson, Malin Persson, Nabil Shalabi, Dilson E. Rassier

**Affiliations:** 1 Department of Chemistry and Biomedical Sciences, Linnaeus University, Kalmar, Sweden; 2 Department of Kinesiology and Physical Education, McGill University, Montreal, Canada; 3 Department of Physiology and Pharmacology, Karolinska Institutet, Stockholm, Sweden

## Abstract

Cyclic interactions between myosin II motor domains and actin filaments that are powered by turnover of ATP underlie muscle contraction and have key roles in motility of nonmuscle cells. The elastic characteristics of actin-myosin cross-bridges are central in the force-generating process, and disturbances in these properties may lead to disease. Although the prevailing paradigm is that the cross-bridge elasticity is linear (Hookean), recent single-molecule studies suggest otherwise. Despite convincing evidence for substantial nonlinearity of the cross-bridge elasticity in the single-molecule work, this finding has had limited influence on muscle physiology and physiology of other ordered cellular actin-myosin ensembles. Here, we use a biophysical modeling approach to close the gap between single molecules and physiology. The model is used for analysis of available experimental results in the light of possible nonlinearity of the cross-bridge elasticity. We consider results obtained both under rigor conditions (in the absence of ATP) and during active muscle contraction. Our results suggest that a wide range of experimental findings from mechanical experiments on muscle cells are consistent with nonlinear actin-myosin elasticity similar to that previously found in single molecules. Indeed, the introduction of nonlinear cross-bridge elasticity into the model improves the reproduction of key experimental results and eliminates the need for force dependence of the ATP-induced detachment rate, consistent with observations in other single-molecule studies. The findings have significant implications for the understanding of key features of actin-myosin-based production of force and motion in living cells, particularly in muscle, and for the interpretation of experimental results that rely on stiffness measurements on cells or myofibrils.

## Introduction

Muscle contraction and several aspects of nonmuscular cell motion are due to cyclic interactions between ensembles of myosin II motors and actin filaments driven by the turnover of ATP (1, 2). The idea that elastic elements in the force-generating cross-bridges between myosin and actin are central for effective production of force and motion dates back several decades (3, 4, 5, 6, 7). The existence of the elastic elements has been verified in experimental studies from both muscle cells (4, 8, 9, 10, 11) and single molecules (12, 13), and the elasticity has key roles in recent models of actin-myosin based contractility (14, 15, 16, 17, 18, 19, 20, 21). Although the cross-bridge elasticity is generally assumed to be linear (Hookean; e.g., (3, 4, 10, 22)), this idea was challenged by experimental results from skinned muscle fibers (23, 24) more than 20 years ago. Recently, the idea of nonlinear cross-bridge elasticity was taken up again based on theoretical considerations (25). It was inferred that there may be buckling of the subfragment 2 (S2) domain between the myosin motor domain and the thick filament backbone when cross-bridges are brought into conformations with negative strain that resist muscle shortening. In contrast, the stiffness of cross-bridges with positive strain is likely (26) to arise from bending of the lever arm (light-chain-binding region) or the nearby converter domain (27, 28). Convincing experimental evidence (29) for appreciably nonlinear cross-bridge elasticity with the proposed structural foundations (25, 26, 27) was recently presented in optical-tweezers-based studies of single molecules. However, these new findings have not noticeably influenced the conception of actomyosin cross-bridge properties in cells (20, 30), and it is by no means self-evident that nonlinearity of the cross-bridge elasticity in muscle cells follows from the single-molecule results. Thus, the ordered myofilament lattice, with short interfilament distances and the presence of numerous accessory proteins (e.g., titin and myosin-binding protein C) may stiffen the S2 region of the myosin molecule and/or prevent it from swinging out from the thick filament backbone. Both these effects may prevent S2 buckling. In agreement with this view, some authors have reported data suggesting linear cross-bridge (and myofilament) elasticity (30, 31). However, several other studies suggest that the myofilaments (32, 33, 34, 35, 36, 37) and/or the cross-bridges (24) exhibit nonlinear elasticity. It is of critical importance to clarify these issues for full understanding of muscle function in health and disease (cf. (27)). Furthermore, if the elastic properties in single-molecule studies differ from those of the cross-bridges in muscle, this has implications for understanding the constraining effects of the ordered myofilament lattice and/or accessory proteins in the muscle sarcomere (38). Before more detailed investigations, the first step is to clarify whether the characteristics of the cross-bridge elasticity in muscle really differ from those found in single molecules.

The previous results, consistent with nonlinear cross-bridge elasticity, have been primarily obtained under rigor conditions, i.e., in the absence of MgATP, in single molecules (29), or in skinned (membrane-free) muscle cells (24, 39). This opens the argument that, although the cross-bridge elasticity may be nonlinear under rigor conditions, the situation may very well be different during active contraction. Furthermore, because of lack of detailed modeling of the muscle fiber data under rigor conditions, it is not clear whether the nonlinearity found previously (24) is consistent with the nonlinearity observed in single molecules (29). An approach to address these questions is to use “bottom-up” modeling (38), i.e., to incorporate the nonlinear cross-bridge elastic properties from single-molecule data (29) into appropriate statistical cross-bridge models (5) for muscle and then test whether these models account for the muscle properties in rigor as well as under physiological conditions.

Here, we perform such studies, using an expanded version of the equilibrium cross-bridge model of Schoenberg (40) for rigor fibers and a minimally modified version of a recent model (18) for active contraction. Our findings corroborate the hypothesis that actomyosin cross-bridges in muscle cells, both in rigor and during active contraction, exhibit nonlinear elasticity similar to that observed in single molecules (29). The physiological importance of nonlinear cross-bridge elasticity is discussed, and further experimental tests are proposed. Full insight into the issue has profound implications for the understanding of actin-myosin based production of force and motion (e.g., (20, 21, 41, 42)) in both health and disease, as well as for the interpretation of experimental data from cellular preparations (10, 33, 34, 38).

## Materials and Methods

### Details of model for rigor conditions

**Figure 1:**
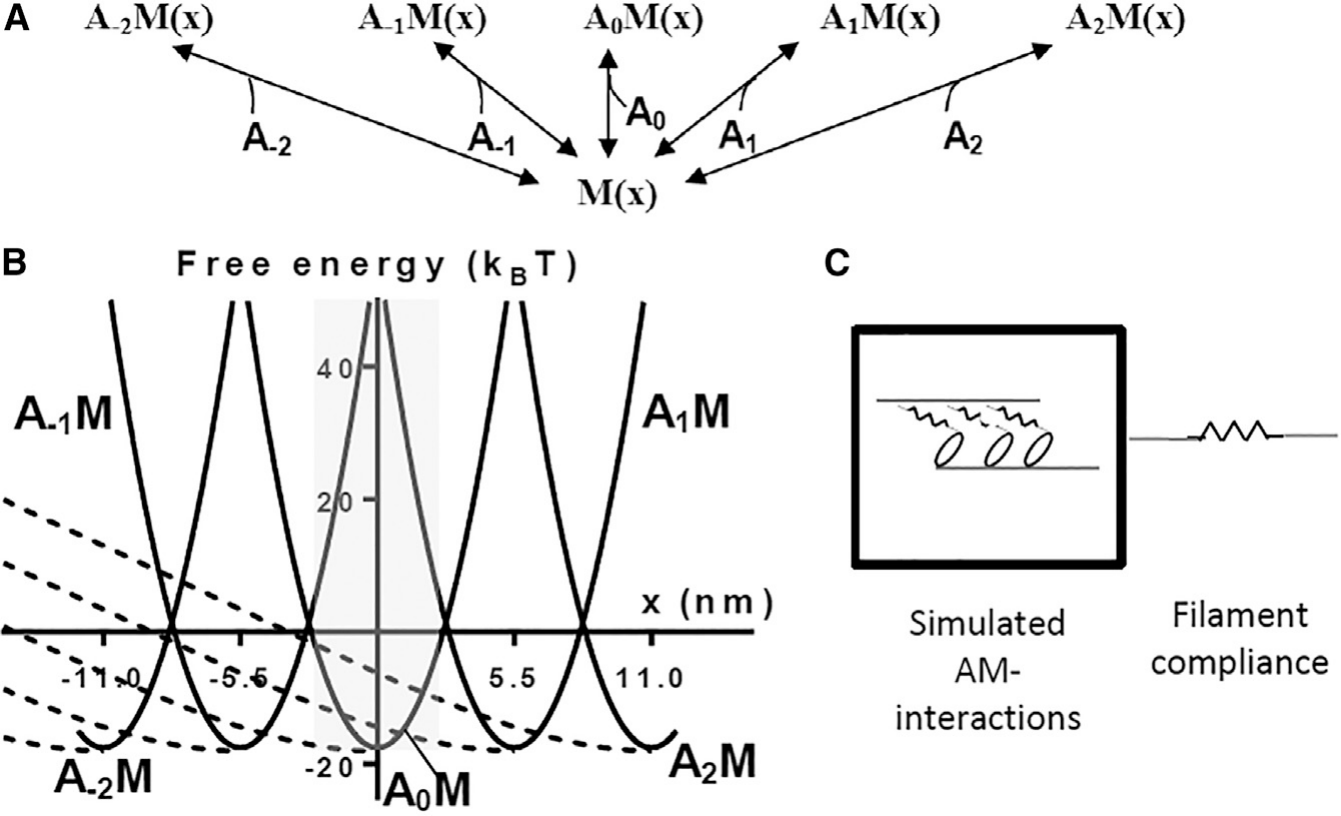
Basic characteristics of model for rigor conditions. (*A*) A kinetic scheme for model with five actin sites (A_−2_…A_2_; separated by 5.5 nm) assumed to be accessible and partly compete for the binding of each myosin head (M) to form rigor (A_*i*_M; actomyosin) links. (*B*) Free-energy diagrams for model states in which free energy of the detached state M is 0 *k*_*B*_*T*. Full curved lines: free energies of A_*i*_M-states in the case of linear (Hookean) cross-bridge elasticity. Dashed curved lines: free energies of A_*i*_M-states in the case of nonlinear cross-bridge elasticity (Fig. S1). The gray area indicates the region for averaging and solving of differential equations to calculate force and stiffness. (*C*) Structural components and their representations in the form of contractile and series elastic elements in the modeling. The series elastic component is limited to myofilament compliance if muscle fiber experiments use sarcomere length control. The myofilament compliance is assumed to be linear and modeled by a separate series elastic element.

We modified the equilibrium cross-bridge model of Schoenberg (40) for the analysis of actomyosin cross-bridge kinetics and the force response to length changes under rigor conditions. Five neighboring sites along the actin filament (A_−2_…A_2_; separated by 5.5 nm) are assumed to be accessible and partly compete for the binding of each myosin head (M) to form rigor (A_*i*_M; actomyosin) links (Fig. 1 *A*). We make the simplifying assumption (40) that the minimal free-energy level (*ΔG*_*AM*_^*min, i*^) of the A_*i*_M state is identical (Fig. 1 *B*) for the different sites (*i* = −2, −1, 0, 1, 2) and equal to −18 *k*_*B*_*T*, i.e., 18 *k*_*B*_*T* below the free energy of the M state ((20); see also (29) and Discussion; 1 *k*_*B*_*T* ≈ 4 pN nm). Here, *k*_*B*_ is the Boltzmann constant, *T* is the absolute temperature, and *i* is an index. The free-energy diagrams for the different A_*i*_ states are displaced by 5.5 nm relative to each other (Fig. 1 *B*) along the filaments reflecting the 5.5 nm spacing between neighboring myosin-binding sites on actin. With Hookean cross-bridge elasticity, the free energy of each A_*i*_M state (1) varies parabolically around the minimal value (5, 6) occurring at *x* = 5.5*i* (*i* = −2…2; scheme 1). We approximate nonlinear cross-bridge elasticity by assuming that the force-extension relationship (*F*(*x* − 5.5*i*)) of the A_*i*_M state is linear for *x* > 5.5*i* (with stiffness *k*_*c*_ = 2.5 pN/nm) and given by a third-order polynomial approximation of the experimental force-extension curve (*F*_*KH*_(*x* − 5.5*i*)) of Kaya and Higuchi (29) for *x* ≤ 5.5*i* (*red line* in Fig. S1).

The detachment rate constant, $k_{d}^{i}$, as a function of *x* has been derived in optical tweezers experiments (43). Adapting these results to our conditions gives

(1) $$k_{d}^{i}\left(x\right)=k_{d}^{i}\left(5.5i\right)\exp\left(\frac{\left|F\left(x-5.5i\right)\right|x_{crit}^{R}}{k_{B}T}\right),$$

with $k_{d}^{i}$ (5.5*i*) set to 0.0014 s^−1^ and *x*_*crit*_^*R*^ = 1.4 nm, respectively. These values correspond approximately to those attributed to two-headed attachment of the heavy meromyosin (HMM) heads to actin (43). The reversal, $k_{a}^{i}$, of the detachment rate constant for each value of *x* has a very high value because of the high affinity between actin and myosin in the rigor state. It is therefore not readily measurable but is given by the following expression:

(2) $$K_{A}^{i}\left(x\right)=k_{a}^{i}\left(x\right)/k_{d}^{i}\left(x\right)=\exp\left(-\Delta G_{AM}^{i}\left(x\right)\right).$$

Here, *ΔG*^*i*^_*AM*_(*x*) is the free-energy difference (in units of *k*_*B*_*T*) between the detached (M and A) and attached (AM) state. It is given by $\Delta G_{AM}^{i}\left(x\right)=\Delta G_{AM}^{\min,i}+1/k_{B}T\int_{5.5i}^{x-5.5i}F\left(x-5.5i\right)dx$ for all *i*. For the case of linear cross-bridge elasticity, Eq. 1 inserted into Eq. 2 gives an attachment rate function $k_{a}^{i}$ (*x*) that is symmetrical around *x* = 5.5*i* (*i* = −2, .., 2). In contrast to the usual Gaussian form of the attachment rate function (5, 19, 40), this function has two symmetrical peaks around *x* = 5.5*i* (for each *i*). Importantly, however, the tension response is primarily determined by $k_{d}^{i}\left(x\right)$ if $k_{a}^{i}$(*x*) ≫ $k_{d}^{i}\left(x\right)$ (40). In these simulations, the latter condition applies for a similar range of *x*-values as for a Gaussian function of similar width.

The cross-bridge stiffness at positive strain (*x* > 5.5*i* for the A_*i*_M state) is taken as 2.5 pN/nm, somewhat lower than the 2.6–2.9 pN/nm estimated in (29) but higher than the 1.7–1.8 pN/nm in other studies (12, 44). For cross-bridges that resist shortening (negatively strained cross-bridges), stiffness is also taken as 2.5 pN/nm in the linear case but is given by $S\left(x\right)=d/dx\left(F_{KH}\left(x-5.5i\right)\right)$, with *F*_*KH*_(*x* − 5.5*i*) defined as described by the third-order polynomial in Fig. S1 *A* in the nonlinear case unless otherwise stated.

To obtain time courses of myosin-head distributions in different states, the following differential equations were solved repeatedly over short time intervals (*Δt*) at 1100 discrete *x*-values in the range *x* = [−2.75, 2.75] nm for all *i*−values from *i* = −2 to *i* = 2:

(3) $$\frac{dm\left(x,t\right)}{dt}=\sum_{i=-2}^{2}\left(a_{i}m\left(x,t\right)k_{d}^{i}\left(x\right)\right)-m\left(x,t\right)\sum_{i=-2}^{2}k_{a}^{i}\left(\text{x}\right),$$

(4) $$\frac{da_{i}m\left(x,t\right))}{dt}=k_{a}^{i}\left(x\right)m\left(x,t\right)-k_{d}^{i}a_{i}m\left(x,t\right).$$

Here, *m*(*x*,*t*) and *a*_*i*_*m*(*x*,*t*) represent the probabilities that the available myosin heads at position *x* and time *t* are in state M and A_*i*_M, respectively. The time intervals *Δt* varied inversely with the speed of the length change (1–1000 nm/s) in the range 0.1–500 ms. After solving the equations in the time interval *Δt* for 1100 discrete *x*-values in the range: [−2.75, 2.75] nm, average cross-bridge force <*F*> and stiffness <*S*> were calculated as follows (5, 40):

(5) $$\langle F\rangle =294\frac{\int_{-2.75}^{2.75}\sum_{i=-1}^{2}a_{i}m\left(x,t\right)(F\left(x-5.5i\right)\;dx}{\int_{-2.75}^{2.75}\left(m\left(x,t\right)+\sum_{i=-1}^{2}a_{i}m\left(x,t\right)\right)dx},$$

(6) $$\langle S\rangle =294\frac{\int_{-2.75}^{2.75}\sum_{i=-1}^{2}\frac{d}{dx}\left(F\left(x-5.5i\right)\right)a_{i}m\left(x,t\right)dx}{\int_{-2.75}^{2.75}\left(m\left(x,t\right)+\sum_{i=-1}^{2}a_{i}m\left(x,t\right)\right)dx}.$$

The expressions contain the multiplier 294 (number of myosin heads per half thick filament) (45)) to give the average force and stiffness per half thick filament. These values are transformed to stiffness and force per cross-sectional area of a muscle cell or myofibril by dividing <*F*> and <*S*>, respectively, with the hexagonal cross-sectional area delimited by six actin filaments with the myosin filament in the center (here taken as 2 × 10^−15^ m^−2^ (cf. (46)).

After calculation of <*F*> and <*S*>, length changes, *Δx*, were imposed to adjust the cross-bridge strain to match the tension in the series elastic elements:

(7) $$\Delta x=\left(k_{se}x_{se}-\langle F\rangle \right)/\left(k_{se}+\langle S\rangle \right).$$

Here, *k*_*se*_ and *x*_*se*_ are the stiffness and strain of a lumped series elastic element attributed to the myofilament compliance (Fig. 1 *C*). An externally imposed length change was first subdivided between the active cross-bridges and the series elastic elements in accordance with the compliance of the two elements given by 1/<*S*> and 1/*k*_*se*_, respectively. Here, the total series elasticity was attributed to the myofilament compliance with stiffness *k*_*se*_ = 150–250 pN/nm corresponding to 75–125 kPa/nm used for the case of nonlinear cross-bridge elasticity. For the case of linear cross-bridge elasticity we used *k*_*se*_ = 250 pN/nm. With these numerical values, ∼70% of the sarcomere compliance at a tension level corresponding to that at isometric contraction would reside in the myofilaments as found experimentally (39).

The model was implemented in MATLAB (The MathWorks, Natick, MA) and solved numerically by the variable order differential equation solver “Ode15s,” based on numerical differentiation formulas. The integrals in Eqs. 5 and 6 were evaluated numerically using the trapezoidal rule.

### Model for active contraction

To evaluate the effect of nonlinear cross-bridge elasticity on active contraction, we limited the studies to steady-state properties, largely eliminating complications due to any nonlinear series elastic components. In the simulations, we used a model (18) that has already been described and tested in detail for the case of linear cross-bridge elasticity. The states in the model as well as the parameter values used have strong support in independent experimental data (18, 38, 47) (see further below). The model in (18) was used in its original form with a cross-bridge stiffness of 2.8 pN/nm (for the linear case) and a free energy of the AM state that is 20 k_B_T lower than for the detached state. These values differ slightly from those (2.5 pN/nm and 18 k_B_T) used for the simulation of rigor conditions above. The differences are consistent with uncertainties in the exact values of the parameters (38) and are unlikely to affect any of the major conclusions of the study. In contrast to the model for rigor fibers, the model for active contraction is of the one-site type, i.e., one myosin head can only reach and bind to one actin site. The effects of making this simplifying assumption have been justified previously (18, 20, 48). The situation is different in rigor conditions because of the high affinity of myosin to actin.

**Figure 2:**
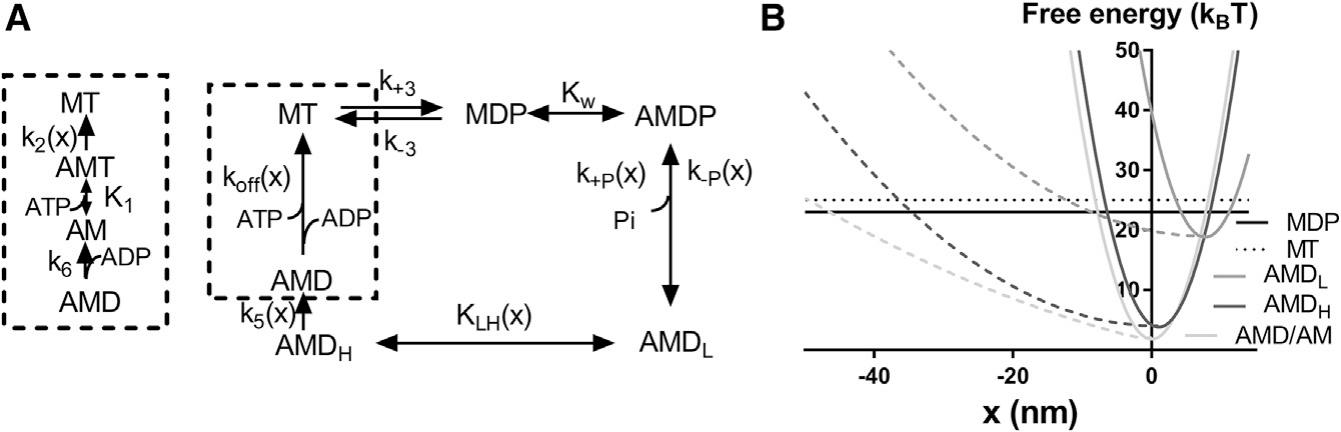
Model for active contraction. (*A*) A kinetic scheme. M, myosin; A, actin; D, ADP; T, ATP; P or P_i_, inorganic phosphate. The constants given by lower case letters represent rate constants. Some of the constants are strain dependent, as indicated by the argument (*x*). Constants *K*_*i*_ represent equilibrium constants. The MDP and the AMDP states are in rapid equilibrium. (*B*) Free-energy diagrams for the different states in the kinetic scheme for the case with linear cross-bridge elasticity (*full lines*) and nonlinear cross-bridge elasticity (*full lines* connected to *dashed lines*) used as standard below (Table S1). The free energy of the weakly attached AMDP state is not shown. It would be represented by a nearly horizontal line (because of very low stiffness, 2.5 k_B_T below the energy for the MDP state).

The model is defined by the kinetic scheme in Fig. 2 *A*, the free-energy diagrams in Fig. 2 *B*, the parameter values in Tables S1 and S2 (see also (18, 47)), and (8a), (8b), (9a), (9b), (10a), (10b), (11a), (11b), (11c), (11d), (11e), 12, 13, 14, 15, 16, 17, (18a), (18b), 19, 20, 21, 22, 23, 24, and 25 below. In the equations, the numerical values of the free-energy differences (*ΔG*_*w*_, *ΔG*_*AMDP-AMDL*_, *ΔG*_*AMDL-AMDH*_, and *ΔG*_*AMDH-AM*_) are given in units of k_B_T, whereas force is given in pN and distances (*x*) in nm.

The equilibrium constant for weak binding of a myosin head to actin is given by

(8a) $$K_{w}\left(x\right)=\text{exp}\left(\Delta G_{w}-\left(ksw/2\right){\left(x\text{-}x_{w}\right)}^{2}/k_{B}T\right)\mathrm{if}\;x\in \left[-\text{0}\text{.}55\text{,}16\text{.0}\right]\text{nm,}$$

(8b) $$K_{w}\left(x\right)=0\;\mathrm{for}\;\mathrm{other}\;\mathrm{x-values}\text{.}$$

In these equations, *ksw* is the stiffness of myosin cross-bridges in the AMDP state and *x*_*w*_ is the *x*-value for minimal free energy of binding of the weakly bound state. The range of *x*-values for which *K*_*w*_(*x*) > 0 is not critical for the cross-bridge attachment range in one-site models with linear or slightly nonlinear cross-bridge elasticity. Under these conditions, the latter range is anyway limited by the high elastic energy in the AMD_L_ state (Fig. 2 *B*). However, for the case of appreciably nonlinear cross-bridge elasticity, such a limitation is important (see below). Tentatively, we limited the attachment range to *x* ∈ [−0.55, 16.0] nm.

The stiffnesses of different states—AMD_L_ (*k*_*s*_^*i*^(*x*)) and AMD_H_ (*k*_*s*_^*ii*^(*x*)), as well as AM—lumped together with AMD, (AM/AMD) (*k*_*s*_^*iii*^(*x*)) are constant (2.8 pN/nm) if the cross-bridge elasticity is linear but vary with *x* in the case of nonlinear cross-bridge elasticity:

(9a) $$k_{s}^{i}\left(x\right)=2\text{.}8\text{pN}/\text{nm}\;\text{for}\;x\geq x_{1},$$

(9b) $$k_{s}^{i}\left(x\right)=0.12\mathrm{pN}/\mathrm{nm}\;\mathrm{for}\;x<x_{1},$$

(10a) $$k_{s}^{ii}\left(x\right)=2.8\mathrm{pN}/\mathrm{nm}\;\mathrm{for}\;x\geq x_{2},$$

(10b) $$k_{s}^{ii}\left(x\right)=0.12\mathrm{pN}/\mathrm{nm}\;\mathrm{for}\;x<x_{2},$$

(11a) $$k_{s}^{iii}\left(x\right)=2.8\mathrm{pN}/\mathrm{nm}\;\mathrm{for}\;x\geq x_{3},$$

(11b) $$k_{s}^{iii}\left(x\right)=0.308+c\left(2.8-0.308\right)\mathrm{pN}/\mathrm{nm}\;\mathrm{for}-4\;\mathrm{nm}<x<x_{3},$$

(11c) $$k_{s}^{iii}\left(x\right)=0.0326+c\left(2.8-0.0326\right)\mathrm{pN}/\mathrm{nm}\;\mathrm{for}-75\;\mathrm{nm}\leq x-x_{3}<-4\;\mathrm{nm}\text{,}$$

(11d) $$k_{s}^{iii}\left(x\right)=2.8\mathrm{pN}/\mathrm{nm}\;\mathrm{for}-90\;\mathrm{nm}\leq x-x_{3}<-75\;\mathrm{nm}\text{,}$$

(11e) $$k_{s}^{iii}\left(x\right)=0\;\mathrm{for}\;x-x_{3}<-90\;\mathrm{nm}\left(\mathrm{obligate}\;\mathrm{cross-bridge}\;\mathrm{detachment}\right)\text{.}$$

The parameter *c*, in (11a), (11b), (11c), (11d), (11e), *b* and *c*, varied between 0 and 1 to allow variation of the cross-bridge elasticity between a fully linear case (*c* = 1) and an extreme nonlinear case (*c* = 0) similar to that in (29). The stiffness values with *c* = 0 correspond to the slopes of the black straight lines in Fig. S1 *A*, fitted to data from (29). The stiffness of the other attached states (AMD_L_ and AMD_H_) for *x* < *x*_*i*_ is approximated by the slope of the gray straight line in Fig. S1 *A*. The latter approximation simplified the calculations and is compatible with very low population of the AMD_L_ and AMD_H_ states for *x* − *x*_*i*_ < −30 nm (see, e.g., Fig. 9).

The transition from the AMDP state to a phosphate-free pre-power-stroke AMD_L_ state (Fig. 2) is governed by

(12) $$k_{+P}\left(x\right)=k_{b0}\;\exp\left[\Delta G_{AMDP\text{-}AMDL}-\left(k_{s}^{i}\left(x\right)/2\right){\left(x-x_{1}\right)}^{2}/\left(2k_{B}T\right)+\left(ksw/2\right){\left(x-x_{w}\right)}^{2}/\left(2k_{B}T\right)\right].$$

The reversal of this transition is governed by

(13) $$k_{\text{-}P}\left(x\right)=k_{b}\left(x\right)\left[{\text{P}}_{\text{i}}\right]/\left(K_{C}+\left[{\text{P}}_{\text{i}}\right]\right),$$

where [P_i_] is the concentration of inorganic phosphate, *K*_*C*_ is the phosphate dissociation constant and

(14) $$k_{b}\left(x\right)=k_{b0}\;\exp[\left(k_{s}^{i}\left(x\right)/2\right){\left(x-x_{1}\right)}^{2}/\left(2k_{B}T\right)-\left(ksw/2\right){\left(x-x_{w}\right)}^{2}/\left(2k_{B}T\right)].$$

The power-stroke or tensing step is assumed to be a rapid equilibrium governed by

(15) $$K_{LH}\left(x\right)=k_{LH+}\left(x\right)/k_{LH-}\left(x\right),$$

where

(16) $$k_{LH+}\left(x\right)=k_{LH-}\left(x\right)\exp\left(\Delta G_{AMDL\text{-}AMDH}+k_{s}^{i}\left(x\right){\left(x-x_{1}\right)}^{2}/\left(2k_{B}T\right)\text{-}k_{s}^{ii}\left(x\right){\left(x-x_{2}\right)}^{2}/\left(2k_{B}T\right)\right)$$

and

(17) $$k_{LH\text{-}}\left(x\right)=2000\;s^{-1}.$$

The existence of states similar to AMD_H_ and AMD in the model has independent support from a range of studies (19, 49, 50, 51, 52, 53). The strain-dependent transition from the AMD_H_ state with closed nucleotide pocket to the AMD state with open pocket (49, 50) is given by

(18a) $$k_{5}\left(x\right)=k_{5}\left(x_{2}\right)\exp\left(\Delta G_{AMDH\text{-}AM}+k_{s}^{ii}\left(x\right){\left(x-x_{2}\right)}^{2}/\left(2k_{B}T\right)-G_{AM}\left(x\right))\right),$$

where

(18b) $$G_{AM}\left(x\right)=\left(\frac{1}{k_{B}T}\right)\left|\int_{x}^{x_{3}}F_{AM}\left(x´-x_{3}\right)dx´\right|,$$

where *F*_*AM*_(*x*′ − *x*_3_) is the piecewise linear function given by full black lines in Fig. S1 *A*.

The AMD state is lumped together with the AM and AMT states (cf. (20)) into what we denote as an AM/AMD state. The detachment rate function (*k*_*off*_(*x*)) for the transition from the AMD into the MT state is (on the assumption that [MgADP] = 0 mM) given by (20) (see also (54)):

(19) $$k_{off}\left(x\right)=\frac{k_{2}\left(x\right)k_{6}\left[\text{MgATP}\right]}{\frac{k_{6}}{K_{1}}+\left(k_{2}\left(x\right)+k_{6}\right)\left[\mathrm{MgATP}\right]}=\frac{k_{2}\left(x\right)\left[\mathrm{MgATP}\right]}{\frac{1}{K_{1}}+\frac{k_{2}\left(x\right)}{k_{6}}\left[\mathrm{MgATP}\right]+\left[\mathrm{MgATP}\right]},$$

where

(20) $$k_{2}\left(x\right)=k_{2}\left(0\right)\exp\left(\frac{\left|F_{AM}\left(x-x_{3}\right)\right|\times x_{crit}}{k_{B}T}\right).$$

In Eqs. 19 and 20, the constants *k*_2_(0) and *k*_6_ govern ATP-induced detachment from the AMT state at *x* = 0 nm and ADP release from the AMD state, respectively. The quantity *K*_*1*_ is the equilibrium constant for MgATP binding to the AM/AMD state (Fig. 2 *A*), and *x*_*crit*_ is a Bell-type strain parameter (55).

To ensure stability in the numerical computations, the value of any rate function (Eqs. 1, 2, 3, 4, 5, 6, 7, (8a), (8b), (9a), (9b), (10a), (10b), (11a), (11b), (11c), (11d), (11e), 12, 13, 14, 15, 16, 17, (18a), (18b), 19, and 20) was limited to a maximum (*r*_max_) of 100,000 s^−1^ for isometric contraction and 1,000,000 s^−1^ for the fastest velocities of shortening and a minimum (*r*_min_) of 1 × 10^−6^ s^−1^. If any of the limits was crossed for a certain value of *x*, the parameter value was set to either *r*_max_ or *r*_min_.

Steady-state contraction with constant velocity, *v*, was simulated under different conditions based on solution of differential equations for the state probabilities (for all *j*,*k*):

(21) $$\frac{da_{j}}{dx}=\left(\sum_{k}^{n1}k_{kj}\left(x\right)a_{k}\left(x\right)-\sum_{k}^{n2}k_{jk}\left(x\right)a_{j\left(x\right)}\right)/v,$$

where *a*_*j*_(*x*) are the state probabilities for the MT (*j* = 4), MDP (*j* = 5), AMD_L_ (*j* = 1), AMD_H_ (*j* = 2), and the AM/AMD (*j* = 3) states in Fig. 2. The rate functions *k*_*kj*_(*x*) and *k*_*jk*_(*x*) represent transitions into state *j* from *n*1 neighboring states and out of state *j* (into *n*2 other states), respectively. The model simulations were implemented by numeric solution of the master equations (Eq. 21), followed by calculation of observable parameters (force and ATP turnover rate) from appropriate state probabilities (48) by averaging over the intersite distance (36 nm) along the actin filament. Thus, average force *<F>* (in pN) per myosin head (whether attached to actin or not) is calculated as

(22) 〈F〉=∑13∫−9114ks(x)aj(x)(x−xj)dx∑15∫−2214aj(x)dx,

whereas the stiffness (〈S〉;Eq. 24) and the fraction of attached myosin heads (<*Na*>; Eq. 25) are obtained as follows:

(23) 〈S〉=∑13∫−9114ks(x)aj(x)dx∑15∫−2214aj(x)dx,

(24) 〈Na〉=∑13∫−9114aj(x)dx∑15∫−2214aj(x)dx.

The quantity *x* is in nm, and *k*_*s*_(*x*) takes any of the values from (9a), (9b), (10a), (10b), and (11a), (11b), (11c), (11d), (11e) as appropriate. Finally, the denominators represent summing over all states and *x*-values in Fig. 2.

The ATP turnover rate (<*ATPase*>) is obtained as follows:

(25) $$\langle ATPase\rangle =\int_{-91}^{14}k_{off}a_{3}\left(x\right)dx\;/\sum_{i}^{5}\int_{-22}^{14}a_{j}\left(x\right)dx.$$

Numerical integration of Eqs. 21, 22, 23, 24, and 25 starts at *x* = 14 nm and progresses in the negative *x*-direction. At *x* = 14 nm, the initial values for all attached states are set to zero, whereas the equilibrium distribution is assumed for the MT and MDP states. The values of the integration limits in Eqs. 22, 23, 24, and 25, as well as the assumptions for the initial values, deserve comments in relation to the one-site model with one myosin-binding site on actin per 36 nm filament half-repeat. The population of the AMD_L_ and the AMD_H_ states is very low for *x* < −22 nm independent of velocity, nonlinearity of cross-bridge stiffness, or other conditions tested here (Fig. S2 *A*). However, because some cross-bridges stay attached in the AM/AMD state for a sliding distance appreciably greater than 36 nm with nonlinear cross-bridge elasticity (Fig. S2 *A*), integration down to *x* = −91 nm is used. Although the one-site approximation is not formally correct under these conditions, it does not produce significantly different results than a formally more correct but appreciably slower method, described in the Supporting Materials and Methods (in relation to Fig. S2). Control simulations using this approach (denoted “periodic boundary conditions”) are reported throughout this manuscript.

### Experimental data from the literature

Experimental data from the literature were obtained by copying relevant figures from cited articles, with subsequent measurements using Image J (56).

## Results

### Rigor conditions: General observations

**Figure 3:**
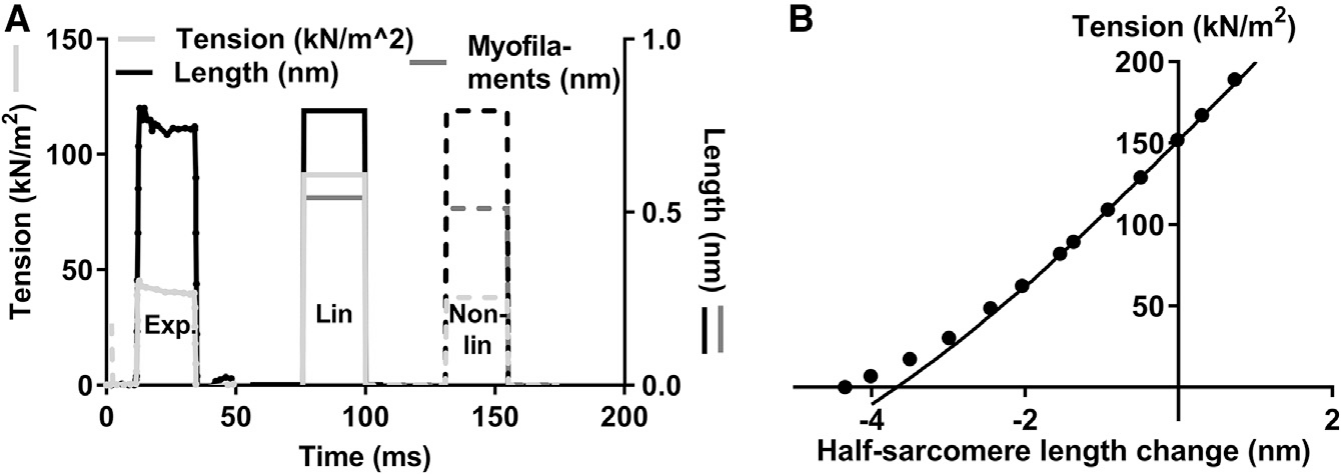
Tension responses to half-sarcomere length changes imposed on a muscle fiber in rigor. A comparison of model responses to experimental data from a skinned rabbit psoas muscle fiber at sarcomere length (2.37 *μ*m) with maximal overlap between thin and thick filaments (39) is shown. (*A*) Tension change (*light gray*; *left vertical axis*) in response to total half-sarcomere-length changes (*black*; *right vertical axis*). Experimental data (*filled circles and lines*; “Exp”), simulated data assuming linear cross-bridge elasticity (*filled straight lines*; “Lin”) or simulated data assuming nonlinear cross-bridge elasticity (*dashed lines*; “Non-lin”) are shown. The cross-bridge force-extension relation for nonlinear case is taken from red line in Fig. S1*A*. The length change attributed to series elastic element in myofilaments (*dark gray*; *right axis*) is also indicated in model simulations. (*B*) The relationship between force and half-sarcomere length (“T_1_ curve”) in experiments (*full circles*) and model simulations (*line*) assuming nonlinear cross-bridge elasticity. The experimental data were obtained by measurements from Fig. 9 *a* in (39)). In the model simulations, ∼1/3 of the total sarcomere compliance was attributed to the cross-bridges, whereas ∼2/3 was attributed to the myofilaments in series (39).

The effects of nonlinear cross-bridge elasticity (29) on the mechanical properties of a muscle fiber in rigor were investigated using the model in Fig. 1. Unless otherwise stated below, we tested aspects of this model under the assumptions of linear myofilament elasticity (see Discussion) and the absence of compliant components outside the sarcomeres. In Fig. 3, model responses, either assuming linear or nonlinear cross-bridge elasticity, are compared to experimental data (39) from a skinned rabbit skeletal muscle fiber in rigor. The time course of the tension change (*ΔT*) closely follows that of the sarcomere length change (*ΔL*) for both models and experiment. Assuming linear cross-bridge elasticity, the model predicts twofold higher sarcomere stiffness (((*ΔT*/*A*)/*ΔL*; A:cross-sectional area) than seen in experiments. This is indicated in Fig. 3 *A* by twofold higher modeled tension response (*full light gray lines*) to a sarcomere length change of given amplitude than in experiments. In contrast, when nonlinear cross-bridge elasticity is assumed (*dashed lines* in Fig. 3 *A*), the model predicts a sarcomere stiffness of 47 kPa/nm, in good agreement with the experimental range (30–70 kPa/nm (24, 31, 39, 44)). Furthermore, the model with nonlinear cross-bridge stiffness faithfully reproduces the experimental relationship (39) between changes in sarcomere length and tension (Fig. 3 *B*) for the tension range between zero and 200 kN/m^2^. The latter value is higher than active isometric tension (150 kN/m^2^) in the same study (39).

**Figure 4:**
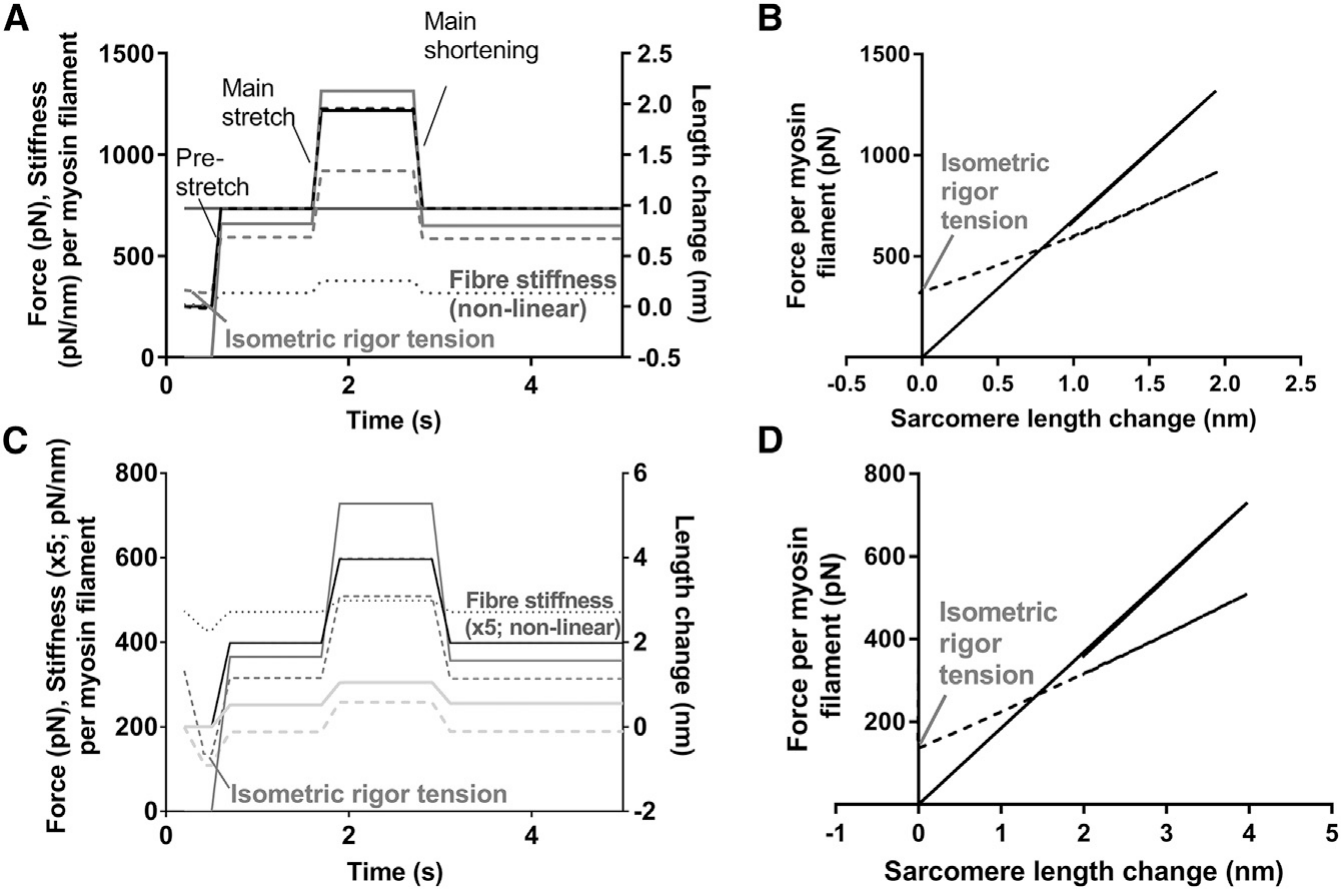
Model predictions for force and stiffness changes upon sequence of stretch-stretch-shortening ramps. (*A*) Time courses with all compliance assumed to reside in cross-bridges. Full lines: linear cross-bridge elasticity. Dashed/dotted lines: nonlinear cross-bridge elasticity. Black: sarcomere length (*right vertical axis*). Gray full and dashed lines: force (*left axis*). Gray, dotted lines: stiffness (*left axis*). Terminology: “prestretch,” “main stretch,” and “main shortening” used regularly in the text. Note nonzero force (“isometric rigor tension”) before any applied length change in the case with nonlinear cross-bridge elasticity. (*B*) Force-sarcomere-length relationships for data in (*A*). Full lines: linear cross-bridge elasticity. Dashed lines: nonlinear cross-bridge elasticity. (*C*) Time courses with 30 and 70% of sarcomere compliance assumed to reside in cross-bridges and myofilaments, respectively. Full lines: linear cross-bridge elasticity. Dashed/dotted lines: nonlinear cross-bridge elasticity. Black: sarcomere length (*right vertical axis*). Light gray: average length change of cross-bridges (*right vertical axis*). Dark gray full and dashed lines: force (*left axis*). Dotted gray line: sarcomere stiffness (*×*5) with nonlinear cross-bridge elasticity (*left axis*). Sarcomere stiffness for linear cross-bridge elasticity is constant and not shown. Note the lower magnitude of the average cross-bridge stiffness with nonlinear compared with linear cross-bridge elasticity. Note also that nonzero force is observed before any applied length changes in the case with nonlinear cross-bridge elasticity. (*D*) Force-sarcomere-length relationships for the data in (*C*). Full lines: linear cross-bridge elasticity. Dashed lines: nonlinear cross-bridge elasticity. Conversion: 200 pN corresponds to 100 kPa for a muscle cell. The average force or stiffness per cross-bridge can be obtained by dividing the values in the figure with 294 (see Materials and Methods).

Our model predicts (Fig. 4) zero isometric rigor tension if the cross-bridge elasticity is linear, but a tension level of 68 kPa (45% of active tension) for nonlinear cross-bridge elasticity. Although it is possible that the experimentally observed rigor tension is a nonequilibrium phenomenon associated with transition into rigor, it is of interest to note that its magnitude (30 ± 4 kPa (33% of active tension) in (24) is within a factor of 2 from the model prediction.

### Toward optimal experimental design for evaluating cross-bridge elasticity in rigor

**Figure 5:**
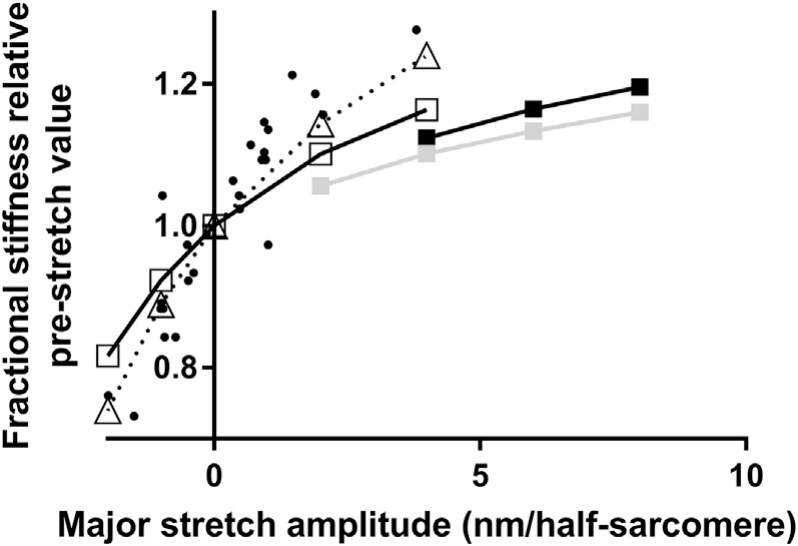
Half-sarcomere stiffness upon stretch ramps (main stretch) of different amplitudes normalized to the stiffness before the stretch. Squares and triangles: simulated data on the assumption of nonlinear cross-bridge elasticity and linear myofilament elasticity. The cross-bridge elasticity was assumed similar to that in (29). Model data are given for main stretches imposed 1 s after a prestretch of amplitude 0 nm (*open symbols*), 1 nm (*black squares*), or 2 nm (*gray squares*). For the case of 0 nm prestretch, data were simulated for two different levels of myofilament stiffness: 150 pN/nm (*squares*) and 250 pN/nm (*triangles*). Negative stretch corresponds to shortening. Simulated data are connected by lines for clarity. Velocity of the simulated length changes was 10 nm/s for all cases. A thousandfold increase in velocity did not change the results. The simulated data are superimposed on experimental results from (24) (*small full circles*; measured from their Fig. 5).

Kaya and Higuchi (29) obtained force-distance relationships from single, full-length myosin molecules interacting with single actin filaments in the absence of ATP. Here, we use our model to evaluate an analogous protocol for use with muscle cells or myofibrils in rigor. In the analysis, we first assumed infinitely stiff myofilaments with all compliant elements residing in the cross-bridges (Fig. 4, *A* and *B*). Initially, a prestretch (cf. (39)) was simulated. This was followed by a “main stretch” and subsequently a “main shortening” (Fig. 4 *A*). The stress-strain relationships (Fig. 4 *B*), associated with the time courses in Fig. 4 *A*, become nonlinear both during the lengthening and the subsequent shortening ramp if the cross-bridge elasticity is changed from linear to nonlinear. The nonlinearity of the stress-strain relationship is associated with higher stiffness during the period after the main stretch and lower stiffness after the main shortening (Fig. 4 *A*). Next (Fig. 4, *C* and *D*), we repeated the modeling after introducing linear myofilament compliance of similar magnitude as assumed in Fig. 3. The major changes of the model predictions, compared to the situation without myofilament compliance (Fig. 4, *A* and *B*), are 1) reduced sarcomere stiffness, 2) barely detectable nonlinearity of the force-length relationship in cases with nonlinear cross-bridge elasticity, and 3) smaller but readily detectable changes in sarcomere stiffness after main stretch and main release. The analysis suggests that experiments with full sarcomere length control (corresponding to simulations in Fig. 4, *C* and *D*) should allow detection of nonlinear cross-bridge compliance from sarcomere stiffness before and after a length change (e.g., measured in response to sinusoidal length oscillations (24, 39)). The model (with nonlinear cross-bridge stiffness) predicts that the increase in sarcomere stiffness upon a stretch (applied to a prestretched muscle preparation) becomes progressively higher (Fig. 5) when the total amplitude of the length change (main stretch + prestretch) increases. The increase during the main stretch would also be higher the smaller the prestretch (Fig. 5). Thus, the analysis suggests that nonlinear cross-bridge elasticity would be most readily detected by stiffness changes in response to main length changes of as large an amplitude as possible and with prestretches as small as possible. The ideal situation would be if no prestretch is imposed, a condition fulfilled in a previous study using skinned frog-muscle fibers (24). In Fig. 5, results from this study (24) (*small full circles*) are compared to simulated data. In agreement with experiments, the model (*open symbols* in Fig. 5) predicts that stiffness increases with increasing stretch amplitude and decreases with increasing amplitude of shortening, showing an upwards convex stiffness-length relationship. Better quantitative fit was observed if higher myofilament stiffness was assumed in the model. The latter modification may be reasonable considering that our model parameter values refer to single rabbit psoas molecules (29) or chemically skinned rabbit psoas fibers (39), whereas the experimental data in Fig. 5 (24) are from freeze-dried, skinned frog-muscle fibers.

### Active muscle contraction

It has been suggested (20) that nonlinearity of the cross-bridge elasticity does not prevent faithful reproduction of the force-velocity relationship of muscle cells. Furthermore, improved reproduction of the relationship between [MgATP] and myosin-propelled actin filament velocity was achieved by switching to nonlinear cross-bridge elasticity (20). Finally, with nonlinear cross-bridge elasticity, the high maximal shortening velocity of muscle could be accounted for without assuming strain-dependent ATP-induced detachment. Here, we elucidate these findings in greater detail using a more realistic model than previously.

Specifically, it is important to use a mechanokinetic model, which accounts for critical contractile phenomena without including states and transitions that lack independent support. This applies to the model (18) as justified in detail (18, 47) previously. Particularly, the three states with ADP at the active site (AMD_L_, AMD_H_, and AMD), which have dominant roles in the model (cf. Fig. 2), find strong support in a range of biochemical (50, 53, 57), structural (58, 59), and single-molecule experimental studies. The three states are important because they are required to account for 1) the main force-generating transition (from the AMD_L_ to the AMD_H_ state (4)) and its occurrence after P_i_-release (47, 58), 2) the biphasic shape of the force-velocity relationship (16, 48, 50, 60) (involving all states: AMD_L_, AMD_H_, and AMD), and 3) findings of a strain-dependent transition (AMD_H_ to AMD state) (49, 50, 53) before the actual ADP release, associated with a second, small (∼1 nm) step in single-molecule displacement records (51). There is also evidence that the AMD_H_ to the AMD transition is rate limiting for the ATP turnover rate during isometric contraction (57). In view of the significance of all mentioned states (out of which AMD is lumped together with other states into an “AM/AMD state”), it is important to consider the possibility that any of them (or all) may exhibit nonlinear cross-bridge elasticity.

**Figure 6:**
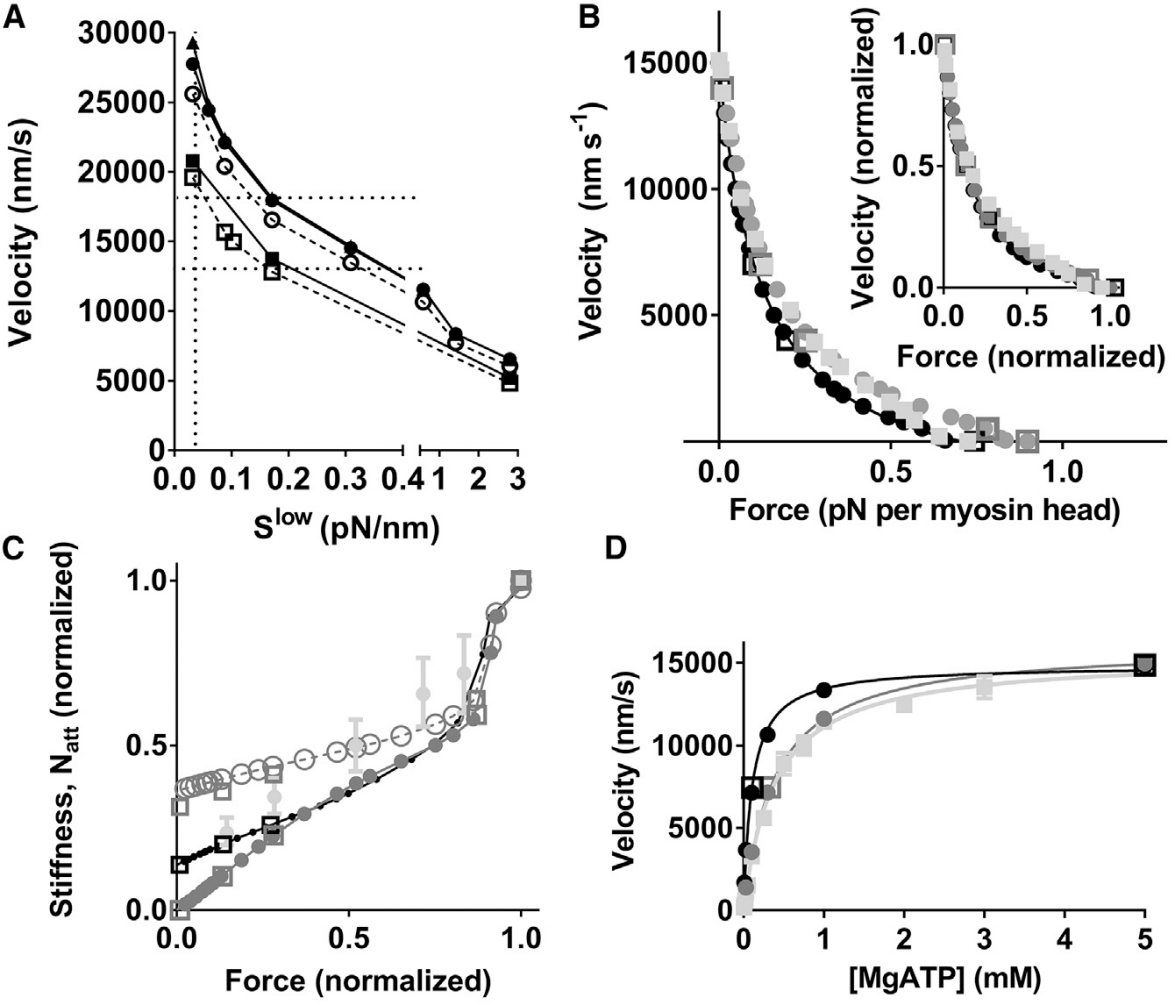
Model predictions for steady-state contractile properties on the assumption of linear or non-linear cross-bridge elasticity. (*A*) Velocity as function of the cross-bridge stiffness (*S*^*low*^) in the AM/AMD state in the range −75 nm < *x* < −4 nm. Rate function *k*_2_(*x*) is strain independent (*x*_*crit*_ = 0). Full lines and filled symbols: *k*_2_(*x*) = *k*_2_(0) = 1800 s^−1^. Dashed lines and open symbols: *k*_2_(*x*) = *k*_2_(0) = 1600 s^−1^. Circles: cross-bridge stiffness linear in AMD_L_ and AMD_H_ states. Filled triangles: stiffness linear in AMD_L_ state but nonlinear in AMD_H_ state ((10a), (10b), *a* and *b*). Filled squares: stiffness linear in AMD_H_ state but nonlinear in AMD_L_ state ((9a), (9b), *a* and *b*). Open squares: nonlinear cross-bridge elasticity in all states. Vertical dotted line: the degree of nonlinearity (*S*^*low*^ = 0.03 pN/nm) observed in AM/AMD state in (29). Horizontal dotted lines: experimental range of velocities (see text for references). (*B*) The force-velocity relationship. The experimental data (*light gray*) are from mouse intact muscle at 30°C (62). Black filled circles: simulations assuming linear elasticity and standard parameter values (Tables S1 and S2) without periodic boundary conditions. Black open squares: similar simulations but with periodic boundary conditions. Dark gray filled circles: simulations assuming nonlinear elasticity and standard parameter values (Tables S1 and S2) without periodic boundary conditions. Dark gray open squares: same as previous but with periodic boundary conditions. Isometric force for experimental data is scaled to data represented by filled black circles. Inset: data normalized to maximal velocity and maximal force for each data set. Note excellent reproduction of experimental data, including nonhyperbolic shape at high loads, by all versions of the model. Maximal isometric force corresponds to ∼120–130 kPa on the whole-fiber level. (*C*) Stiffness attributed to linear cross-bridge elasticity in experimental data (10) (*light gray*), corrected for linear myofilament elasticity estimated in the same experiments. Note that the experiments (mean ± standard error of the mean) are from frog fast muscle fibers at 4°C. The grayscale coding of symbols and lines is the same as in (*B*) but with the addition of open circles representing normalized number of attached cross-bridges (*N*_*att*_) for nonlinear cross-bridge elasticity in all states. Open squares represent simulations of stiffness and *N*_*att*_ using periodic boundary conditions. (*D*) Velocity versus [MgATP] for experimental data (20) and simulations assuming either linear (*black*) or nonlinear (*dark gray*) cross-bridge elasticity for all attached cross-bridge states is shown. The same grayscale coding as in (*B*) is used.

Initially (Fig. 6 *A*), we used the model to investigate the relationship between the degree of nonlinearity of the cross-bridge elasticity and the maximal velocity of shortening if the detachment rate function is assumed to be unaffected by strain (i.e., *k*_2_(*x*) = *k*_2_(0); *x*_*crit*_ = 0 nm; Eq. 20). Preliminary simulations suggested that the elastic properties in the AM/AMD state are most important. Therefore, we varied the force-extension relationship in that state (Fig. 6 *A*; by varying *c* in (11a), (11b), (11c), (11d), (11e), *b* and *c*) between the fully linear case and the degree of nonlinearity in (29). The stiffness value (*S*^*low*^) in the range −75 nm < *x* < −4 nm was taken as an index of the nonlinearity in the AM/AMD state. When *S*^*low*^ is 2.8 pN/nm, the cross-bridge elasticity in this state is linear, whereas *S*^*low*^ = 0.03 pN/nm corresponds to nonlinearity as in (29) (cf. Fig. S1). Only two discrete, alternative, force-extension relationships (linear or nonlinear; (9a), (9b), *a* and *b* and 10, *a* and *b*) were considered for the AMD_L_ and AMD_H_ states. Consideration of the strain dependence of k_2_(*x*) is important because single-molecule (61) studies suggest negligible strain dependence, whereas models with linear cross-bridge elasticity (18, 20) seem to require strain dependence to account for the experimentally observed unloaded shortening velocity of fast mammalian muscle (13,000–18,000 nm/s on filament level; ∼30°C) (62, 63, 64, 65). Accordingly, as shown in Fig. 6 *A*, with *x*_*crit*_ = 0 nm (no strain dependence), the simulated velocity is about half the experimental value if linear cross-bridge elasticity is assumed in the AM/AMD state (*S*^*low*^ = 2.8 pN/nm). The situation is minimally changed (Fig. 6 *A*) by altered elastic characteristics of the AMD_L_ and/or the AMD_H_ state. The velocity reaches the experimentally observed range only if *S*^*low*^ in the AM/AMD state is reduced below 0.34 pN/nm with linear elasticity in the AMD_L_ and AMD_H_ states or nonlinear elasticity in the AMD_L_ (but not AMD_H_) state. If both the AMD_H_ and AMD_L_ states exhibit nonlinear elasticity of a type seen in (29), *S*^*low*^ for the AM/AMD state needs to be reduced below 0.2 pN/nm to reach the experimentally observed velocity range. Interestingly, with the most extreme degree of nonlinear elasticity tested (*S*^*low*^ as in Kaya and Higuchi (29); *vertical dotted line* in Fig. 6 *A*), the simulated velocity is appreciably higher than in experiments. Reduction of *k*_2_(0) from 1800 to 1600 s^−1^ (lowest value within the experimental uncertainty (66)) only minimally improved the situation (Fig. 6 *A*; *dashed lines*, *open circles*). To achieve the experimentally observed velocities, it is necessary to assume that the nonlinearity of the elasticity in the AM/AMD state is less accentuated (*S*^*low*^ ≈ 0.1 pN/nm) than in (29) (*S*^*low*^ ≈ 0.03 pN/nm). Although this value (*S*^*low*^ ≈ 0.1 pN/nm) is more than threefold higher than in (29), it is important to note that it is more than an order of magnitude lower than for linear cross-bridge elasticity (*S*^*low*^ ≈ 2.8 pN/nm).

One may also consider the possibility that the experimentally observed maximal velocity could be explained with linear cross-bridge elasticity without any force dependence of *k*_2_(*x*) (*x*_*crit*_ = 0 nm), simply by increased value of *k*_2_(0). However, to account for the observed maximal velocity (>13,000 nm/s), *k*_2_(0) would need to be increased to at least 6430 s^−1^. This is more than threefold higher than the value observed using isolated myosin from rabbit psoas muscle at 30°C (18, 66). Even if the magnitude of the strain-dependent rate function *k*_5_(*x*) between the AMD_H_ and the AMD state is increased 10-fold, an almost 2.5-fold increase of *k*_2_(0), compared to literature data (18, 66), would be required to achieve a maximal velocity of 13,000 nm/s with linear cross-bridge elasticity and *x*_*crit*_ = 0 nm. In summary, nonlinear cross-bridge elasticity seems necessary to account for the experimentally observed maximal velocity if the ATP-induced detachment rate (*k*_2_(*x*)) is strain independent (*k*_2_(*x*) = *k*_2_(0); *x*_*crit*_ = 0 nm). The modeling suggests that the degree of nonlinearity of the cross-bridge elasticity in muscle cells, although substantial, is smaller than in single molecules.

**Figure 7:**
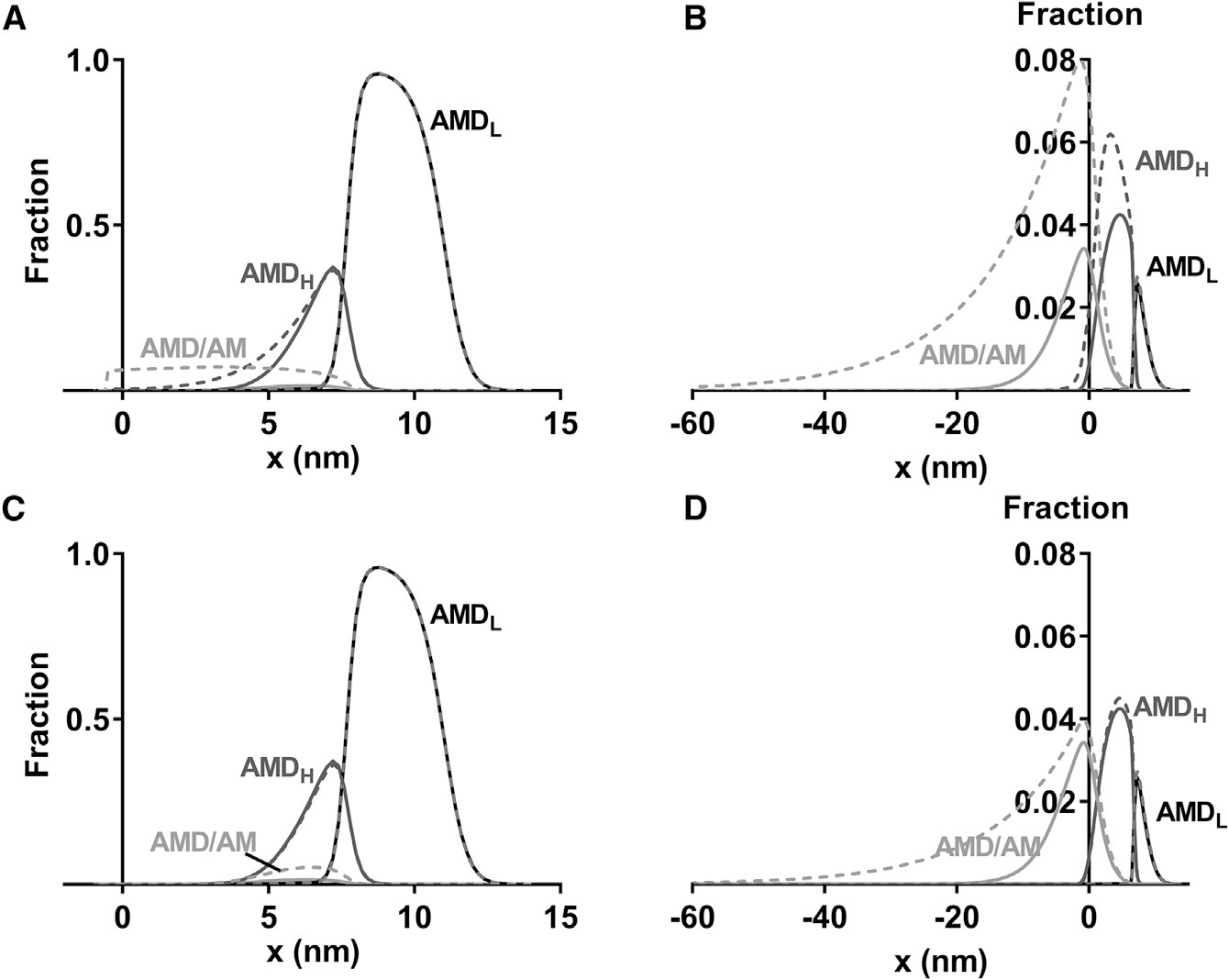
Population of different cross-bridge states during isometric contraction (*left panels*) and during shortening near maximal velocity (*right panels*). (*A*) Cross-bridge distributions during isometric contraction, assuming that all attached cross-bridge states have linear (*full lines*) or nonlinear elasticity (*dashed lines*). (*B*) Cross-bridge distributions as in (*A*) (same grayscale coding) but simulated for steady-state shortening at 14,000 nm/s. (*C*) Cross-bridge distributions during isometric contraction assuming that all attached cross-bridges have linear elasticity (as in *A*; *full lines*) or that the AMD/AM state exhibits nonlinear elasticity, whereas the other states have linear elastic elements (*dashed lines*). (*D*) Cross-bridge distributions during shortening at 14,000 nm/s, assuming either linear cross-bridge elasticity of all states (as in *B*; *full lines*) or nonlinear elasticity of the AM/AMD state only (*dashed lines*). The distributions are normalized to the total number of myosin heads at each value of *x*.

If not otherwise stated below, we assume for the case with nonlinear cross-bridge elasticity that *k*_2_(0) = 1600 s^−1^ (*x*_*crit*_ = 0 nm) and *S*^*low*^ = 0.1 pN/nm in the AM/AMD state (corresponding to *c* = 0.0255 in (11a), (11b), (11c), (11d), (11e), *b* and *c*) and that the AMD_L_ and AMD_H_ states exhibit nonlinear elasticity with stiffness 0.12 pN/nm in the drag-stroke region ((9a), (9b), (10a), (10b)). For the case of linear cross-bridge elasticity, we assume that *k*_2_(0) = 1800 s^−1^ and *x*_*crit*_ = 0.6 nm. Changing the elasticity from linear to nonlinear in the model on these assumptions had negligible effects on the shape of the force-velocity relationship (Fig. 6 *B*, *inset*) but slightly increased the maximal isometric force. In both cases, the fit to experimental data is excellent, as best appreciated from the normalized data in the inset of Fig. 6 *B*. Also, if only the AM/AMD state (and not the AMD_L_ and AMD_H_ states) is assumed to have nonlinear elasticity, the experimental data are well predicted (data not shown). Importantly, the maximal velocity of shortening is similar for the simulations with linear and nonlinear elasticity only because of a suitably chosen strain dependence of the ATP-induced detachment rate (*k*_2_(*x*)) in the linear case (*x*_*crit*_ = 0.6 nm) (18). No such assumption is needed for the case of nonlinear cross-bridge elasticity. Also, the experimental relationship between force and stiffness during steady shortening at different velocities (obtained in frog muscle fibers (10)) is rather well predicted whether linear or nonlinear cross-bridge elasticity is assumed (Fig. 6 *C*). The total number of attached cross-bridges during shortening at increasing velocities (at reduced steady force) is reduced in proportion to the reduction in stiffness if cross-bridge elasticity is linear. In contrast, the reduction in the total number of attached cross-bridges is appreciably smaller than the reduction in stiffness if the cross-bridge elasticity for all states is nonlinear (*open circles* in Figs. 6 *C*; see also Fig. 7). There is also a higher number of attached cross-bridges compared to the linear case during shortening at maximal velocity if only the elasticity of the AM/AMD state is nonlinear. Quantitatively, the number of attached cross-bridges in the latter case is intermediate (∼21% of isometric value) between that for linear cross-bridge elasticity (∼15%) and that for nonlinear cross-bridge elasticity in all states (∼30%). Furthermore, when going from isometric force to zero force, the model predicts that the average cross-bridge strain is reduced from 2.43 to 0 nm in the case of linear cross-bridge elasticity, compared to a reduction from 2.54 to −10.83 nm if the cross-bridge elasticity is nonlinear in all states and 2.54 to −8.89 nm if the cross-bridge elasticity is nonlinear only in the AM/AMD state (cf. cross-bridge distributions versus strain (*x*) in Fig. 7).

**Figure 8:**
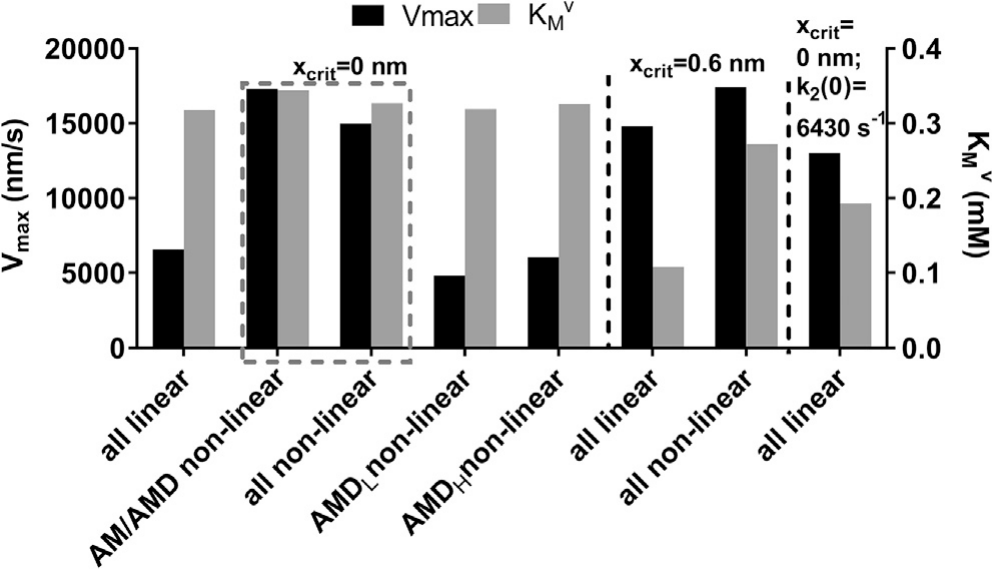
The parameter values *V*_*max*_ and *K*_*M*_^*v*^ for actin filament sliding velocity versus [MgATP]. *V*_*max*_ (*black*, *left axis*) and *K*_*M*_^*v*^ (*gray*, *right axis*) were obtained in simulations with different combinations of linear and nonlinear (from (29)) cross-bridge elasticity for different states and force dependence of *k*_2_(*x*) ((*x*_*crit*_ = 0 nm; no force dependence) or (*x*_*crit*_ = 0.6 nm; force dependence)). The final group of parameter values (*right*) was obtained assuming linear cross-bridge elasticity and ATP-induced detachment rate constant without force dependence, but *k*_2_(0) increased almost threefold to 6430 s^−1^ compared to data in Table S2. The box indicates simulations in which both *V*_*max*_ and *K*_*M*_^*v*^ values accord best with experimental data.

In agreement with previous studies using simpler models or simpler representation of the cross-bridge elasticity (18, 20), we found (Fig. 6 *D*) that the experimental [MgATP]-velocity relationship is more faithfully reproduced if nonlinear cross-bridge elasticity is assumed instead of linear elasticity. The rectangular hyperbolic shape of the relationship and the maximal velocity (*V*_*max*_) are reproduced with both linear (with *x*_*crit*_ = 0.6 nm) and nonlinear (with *x*_*crit*_ = 0 nm) cross-bridge elasticity. However, the experimentally observed (∼0.4 mM (20, 67)) value of the MgATP concentration, *K*_*M*_^*v*^, for half-maximal velocity is well predicted (Fig. 6 *D*) only with nonlinear elasticity and *x*_*crit*_ = 0 nm. It was not clear whether introduction of the nonlinear cross-bridge elasticity or the removal of the force dependence of *k*_2_(*x*) (*x*_*crit*_ = 0 nm) is most important in causing the increase of *K*_*M*_^*v*^ from ∼0.1 to >0.3 mM. Furthermore, it was not clear if the elastic characteristics of only the AM/AMD state or all attached states are important. Results of simulations to elucidate these issues are depicted in Fig. 8. It can be seen (*box* in Fig. 8) that both *V*_*max*_ and *K*_*M*_^*v*^ values approximately consistent with experimentally observed ranges (see above) are achieved for *x*_*crit*_ = 0 nm either if all attached cross-bridge states have nonlinear elasticity or if nonlinear elasticity is assumed only for the AM/AMD state. The high maximal velocity of shortening with *x*_*crit*_ = 0 nm is not reproduced if only the AMD_L_ or the AMD_H_ states are assumed to exhibit nonlinear elasticity. However, the experimentally observed high *K*_*M*_^*v*^ value is reproduced for these cases. This led us to hypothesize that a high *K*_*M*_^*v*^ value is attributed to the lack of strain dependence (*x*_*crit*_ = 0 nm) of *k*_2_(*x*) rather than to the introduction of nonlinear cross-bridge elasticity. Simulation of the case with linear cross-bridge elasticity, assuming *x*_*crit*_ = 0, supports this view (*leftmost bars* in Fig. 8). Thus, although *V*_*max*_ is less than half the *V*_*max*_ value for the case in which all cross-bridge states have nonlinear elasticity, the *K*_*M*_^*v*^ value is quite similar to that obtained on the assumption of nonlinear cross-bridge elasticity. However, it should be noted that lack of strain dependence (with *x*_*crit*_ = 0) is not necessary to account for high *K*_*M*_^*v*^ under all conditions. Thus, introduction of nonlinear cross-bridge elasticity with strain dependence of *k*_2_(*x*) (*x*_crit_ = 0.6 nm) causes *K*_*M*_^*v*^ to increase almost to the same extent as the removal of the strain dependence per se (second and third *gray bars* from the end in Fig. 8). Finally, whereas an almost threefold increase of *k*_2_(0) under the assumption of linear cross-bridge elasticity with *x*_*crit*_ = 0 nm (*rightmost bars* in Fig. 8) could predict *V*_*max*_, the *K*_*M*_^*v*^ value is <0.2 mM. The results in Figs. 6 *D* and 8 can be summarized as follows. First, in a model with linear but not with nonlinear elasticity of AM/AMD cross-bridges, *k*_2_(*x*) must exhibit strain dependence (increase with strain) to account for the maximal velocity of shortening. Second, both removed strain dependence of *k*_2_(*x*) and introduction of nonlinear cross-bridge elasticity per se contribute to increased *K*_*M*_^*v*^ value when switching from a model with linear cross-bridge elasticity and *x*_*crit*_ = 0.6 nm to a model with nonlinear elasticity and *x*_*crit*_ = 0 nm. Finally, nonlinear elasticity of the AM/AMD state is most important in explaining both the high *V*_*max*_ and the *K*_*M*_^*v*^ values for a model with *x*_*crit*_ = 0 nm.

**Figure 9:**
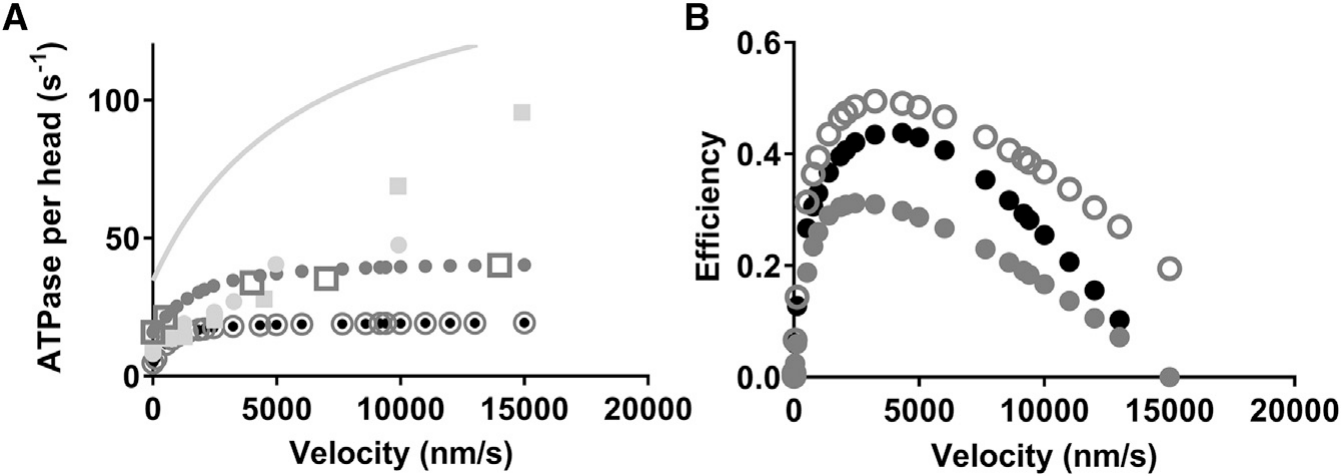
Steady-state ATP turnover rate and thermodynamic efficiency. (*A*) ATP turnover rate per all available (not only attached) myosin heads as a function of sliding velocity on the assumption of linear (*black*) or nonlinear (*dark gray*) cross-bridge elasticity. The nonlinear cross-bridge elasticity was simulated either assuming that the elasticity is nonlinear for all attached states (*filled dark gray circles*) or only for the AM/AMD state (*open gray circles*). Experimental data are illustrated by the light gray line and symbols from (69) (*circles*), (102) (*line*), and (70) (*squares*). The velocity data were transformed from the original experimental values by first normalizing velocity to the unloaded velocity in each experiment and then scaling to the high-temperature value of our simulations. The ATP turnover rate was transformed by assuming *Q*_*10*_ = 2.9 for ATP turnover rate at all velocities. For more details, see text. All simulated data represented by circles were obtained without consideration of periodic boundary conditions. Control simulations using periodic boundary conditions are represented by open squares for nonlinear cross-bridge elasticity. (*B*) Thermodynamic efficiency data calculated from simulated results in Figs. 6*B* and 9*A*. The same grayscale coding as in (*A*) is used.

It is of interest to consider possible evolutionary mechanisms that might have favored the development of nonlinear cross-bridge elasticity, i.e., why would nonlinear cross-bridge elasticity be physiologically advantageous? One possibility (29, 42) is that reduced braking forces during shortening, due to reduced force in negatively strained cross-bridges, could increase efficiency. In both the cases with linear and nonlinear cross-bridge elasticity, and in general agreement with experimental data (68), the model predicts increased ATP turnover rate with increased velocity (Fig. 9 *A*). However, for all velocities, the ATP turnover rate is appreciably higher for the case with nonlinear compared to linear cross-bridge elasticity. This is attributed to an increased number of attached cross-bridges in the case of nonlinear cross-bridge elasticity (Figs. 6 *C* and 7). This, in turn, is due to lowered free energy in the AMD_L_ state for a wide range of *x*-values (*dashed red line* in Fig. 2 *B*), associated with increased equilibrium constant for cross-bridge attachment for these *x*-values. In accordance with this mechanism, increased ATP turnover rate compared to the case with fully linear cross-bridge elasticity is not seen if nonlinearity is assumed only for the cross-bridge elasticity of the AM/AMD state. The latter intervention does not increase the equilibrium constant for attachment. When assuming nonlinear cross-bridge elasticity also in the AMD_L_ state, it is important (see below) to limit the range of *x*-values at which attachment is at all possible. The lower and upper limits used here were −0.55 and 16 nm, respectively. If the lower limit is reduced from −0.55 to −2.8 nm, the simulated maximal velocity is reduced from 20,790 to 19,310 nm/s. Further, the maximal ATP turnover rate is increased, and the maximal efficiency is reduced. Conversely, a narrower attachment range (lower limit increased from −0.55 to 1.7 nm) has the opposite effect. Thus, velocity is, under these conditions, increased from 20,790 to 23,670 nm/s, and the ATP turnover rate is reduced.

There is appreciable variability in experimental data for the ATP turnover rate under isometric conditions (69, 70, 71) as well as for the effect of shortening on this parameter (68, 69, 70, 72, 73, 74). Furthermore, the experimental results have been obtained at different temperatures, and the temperature dependence of maximal velocity (*Q*_*10*_ ≈ 2.1 (66)), isometric ATPase (*Q*_*10*_ ≈ 2.9 (71)), and ATPase at high shortening velocity (no *Q*_*10*_ value found in the literature) seems to differ. In view of these complexities, comparison of the simulated data in Fig. 9 *A* to experimental results is challenging. Tentatively, we assumed *Q*_*10*_ = 2.9 for both isometric and isotonic ATP turnover rates and normalized velocity to the maximal value in each study (cf. (68)) followed by scaling to model data (temperature of 30°C). With this approach, there is fair quantitative agreement between models and experiments considering the variability between different studies and other uncertainties. Interestingly, the experimental ATP turnover data show greater quantitative agreement with the experimental data if all cross-bridge states are assumed to have nonlinear elasticity (Fig. 9 *A*).

By combining the force-velocity data in Fig. 6 *B* with the ATP turnover data in Fig. 9 *A*, it follows that introduction of nonlinear cross-bridge elasticity for all cross-bridge states leads to appreciably reduced thermodynamic efficiency (Fig. 9 *B*). However, in contrast, if nonlinear elasticity is assumed only for the AM/AMD state, the efficiency is increased compared to the linear case because of somewhat higher power output at intermediate loads (Fig. 6 *B*). The model predictions for efficiency are somewhat higher than found experimentally for fast mammalian muscle, but these values vary quite substantially between studies (0.20–0.46) ((68, 70) and references therein), consistent with the uncertainties and variabilities in experimental ATPase data (see above).

Another possible evolutionary advantage of nonlinear compared to linear cross-bridge elasticity, as suggested by modeling, is that more cross-bridges would be attached during fast shortening in the nonlinear case (Fig. 6 *C*). These cross-bridges have both low stiffness and highly negative strain on average (Fig. 7). The implications of these results are discussed in detail below.

## Discussion

### Rigor fibers

Our model for rigor conditions builds on that of Schoenberg (40) but is modified to incorporate recent experimental data for 1) myofilament compliance (39), 2) cross-bridge detachment rates (43), and 3) nonlinear cross-bridge elasticity (29). In similarity to the previous model (40), we assume that detached myosin heads rapidly rebind in the same biochemical state (AM) either to the same or to a neighboring site along the actin filament. Such reattachment to nearby sites is important in the modeling of rigor muscle because of the high actin-affinity of myosin in the nucleotide-free state, making binding likely despite nonoptimal geometry. A difference between muscle and the model is that the neighboring sites in a real muscle are azimuthally rotated relative to each other, as seen by myosin molecules on a given thick filament (40, 75), and/or possibly located on different thin filaments (76). Nevertheless, following previous arguments (40), we assumed the same shape of the free-energy profiles of each site albeit displaced relative to each other along the filament. This is the simplest approximation, and the effects on the free-energy diagrams of the complicating factors are difficult to predict.

The detachment rate as a function of the imposed strain has been explicitly determined for rigor cross-bridges (43) in unbinding studies using optical tweezers. Two different functions were found, attributed to one-headed and two-headed attachments, respectively. Here, we used the function consistent with two-headed attachment (43). The reason is better prediction of a very slow tension decay after an imposed stretch on rigor muscle fibers (cf. (77)) and agreement with evidence that all myosin heads are attached to actin in rigor (78, 79, 80). Because the use of this rate function leads to faithful reproduction of experimental data, we did not test other mechanisms, e.g., slip-catch behavior of the actomyosin bond (81).

In our analysis of rigor fibers, we assumed linear myofilament elasticity in agreement with several (e.g., (10, 24, 31)) but not all (33, 34, 82) studies. Furthermore, the myofilament elasticity was lumped together into a series elastic component, an approach that has been justified previously (83). If the myofilament elasticity is nonlinear, the proposed approach (Figs. 3, 4, and 5) for quantitatively evaluating the possible nonlinearity of the cross-bridge elasticity would not be readily feasible. Such an evaluation would then require that the characteristics of the myofilament elasticity be fully known. Interestingly, the extent of nonlinearity of the experimental force-extension relationship in rigor muscle preparations (39) (Fig. 3 *B*) and the associated strain dependence of stiffness (24) (Fig. 5) are greater than predicted by our model if we assume nonlinearity of the cross-bridge elasticity similar to that found in single molecules (29). The comparably low degree of nonlinearity in the model simulations (Fig. 3 *B*) may seem somewhat surprising in view of the appreciable nonlinearity of the cross-bridge force-extension curve (Fig. S1) (29). However, this can be understood from the fact that the nonlinearity in a muscle fiber is attenuated by an ensemble of cross-bridges at different strains. For similar reasons, the force-extension relation of a rigor fiber, as predicted by the model, intersects the zero-force level without change in slope (Fig. 3 *B*). This is in agreement with what is observed in muscle fiber experiments (22) and seems to invalidate arguments against nonlinear cross-bridge elasticity based on the latter result ((22); argument restated in (30)). The less substantial nonlinearity predicted by the model for rigor fibers than observed in the experiments (Figs. 3 *B* and 5) could mean that the myofilament elasticity is also nonlinear. Alternatively, the cross-bridge elasticity in muscle fibers exhibits even greater nonlinearity than suggested by the single-molecule data or there is substantial slackening of myofilaments at low tension (39). There are arguments against the idea of slackening filaments based on the frequency dependency of stiffness changes with strain (24). Furthermore, our analysis of active steady-state contraction, not expected to be influenced by the characteristics of the myofilament elasticity, seems to suggest a lower degree of nonlinearity of the cross-bridge elasticity than observed by Kaya and Higuchi (29). This argues for nonlinearity of the myofilament elasticity also to account for the data in rigor. In summary, our analysis suggests that the mechanical properties of muscle fibers in rigor are consistent with nonlinear cross-bridge elasticity. The findings are also consistent with nonlinear myofilament elasticity and/or contribution to the observed nonlinearity in muscle preparations by other, yet unidentified, phenomena found previously (30, 84). However, whether the latter phenomena would be important at the presumably constant activation level in rigor fibers is unclear.

### Active contraction

In analysis of active contraction, complexities due to possible nonlinearity of the myofilament elasticity were avoided by focusing on steady-state properties, something that is not possible with rigor fibers. The model for active contraction is taken directly from (18) and only includes states and transitions that are essential to account for key aspects of contractile function (e.g., biphasic force-velocity relationship (60)). The inclusion of the states and transitions has been motivated in detail recently (18, 47) on the basis of evidence from a wide range of independent studies (29, 49, 50, 51, 53, 60, 61, 85, 86, 87, 88). Furthermore, when defining the model (18), care was taken to use parameter values for conditions as coherent as possible with regard to ionic strength (>100 mM), temperature (close to 30°C), and animal species as well as muscle type (fast mammalian muscle). Second, whereas the parameter values were largely derived using experiments on isolated proteins (a “bottom-up” model) (38), the model with linear cross-bridge elasticity accounts for a range of experimental results from the single-molecule level over in vitro motility assays to muscle mechanics (18).

Because the model is of the “bottom-up” type (38), with parameter values fixed on basis of independent literature data, it predicts (rather than fits) experimental data. If the prediction is good, this supports the validity of the parameter values and the model. If not, the model has to be modified or one critical parameter value under study may be modified. Here, we found that a perfect reproduction of experimentally observed maximal velocities was not possible with parameter values defined entirely from the bottom up. We therefore varied the degree of nonlinearity of the cross-bridge elasticity between the fully linear case and the extremely nonlinear case found in single molecules (29). Importantly, the only other change from the independently derived parameter values (Tables S1 and S2) is a reduction in *k*_2_(0) by 10% (within the experimentally observed range).

A discrepancy between the version of our model with linear cross-bridge elasticity (18) and results from isolated fast skeletal muscle myosin ((61); see further (18, 38)) is the need to invoke a force-dependent function *k*_2_(*x*) in the model to reproduce the experimentally observed shortening velocities. Using a value of *k*_2_(0) derived from (66), it was necessary with a force dependence corresponding to the parameter value *x*_*crit*_ = 0.6 nm if the cross-bridge elasticity was assumed to be linear. This is in apparent conflict with a very limited force dependence of the corresponding rate function in single-molecule data (61). As shown in Fig. 6 *A*, appreciable nonlinearity in the cross-bridge elasticity, albeit less than in (29), needs to be assumed to account for the maximal velocity with *x*_*crit*_ = 0 nm. After implementing this required change and assuming nonlinear elasticity for all states, we went on with further tests of the model. First, we found that it accounts very well for the force-velocity data and rather well for force-stiffness data during shortening at different velocities. Finally, the model with nonlinear cross-bridge elasticity and *x*_*crit*_ = 0 nm gives appreciably better predictions for the [MgATP]-velocity relationship than the original model (18) with linear cross-bridge elasticity and *x*_*crit*_ = 0.6 nm.

The relationship between sliding velocity and [MgATP] is characterized by a *K*_*M*_^*v*^ value that increases with temperature (along with increase in *V*_*max*_) in skinned muscle cells (67) from ∼0.15 mM at conventionally used low temperatures (∼10°C) in such experiments to ∼0.6 mM at 35°C, corresponding to *Q*_*10*_ of ∼1.7. These data are in good agreement with the *K*_*M*_^*v*^ value of 0.30 mM found in (89) for thin filaments (actin reconstituted with tropomyosin and troponin) in the in vitro motility assay using HMM adsorbed to nitrocellulose at 25°C. The data are also in good agreement with the results (*K*_*M*_^*v*^ ≈ 0.39 mM) of Persson et al. (20) (experimental data in Fig. 6 *D*) using actin filaments in the in vitro motility assay with HMM adsorbed to trimethylchlorosilane-derivatized surfaces at 28–29°C. The latter data (20) also exhibit similar *V*_*max*_ values as muscle fibers at similar temperatures. Persson et al. (20) also found similar maximal sliding velocity with pure actin filaments and HMM adsorbed to nitrocellulose. This is in contrast to the data for pure actin and HMM in (89, 90) (also using adsorption to nitrocellulose) in which both *V*_*max*_ and *K*_*M*_^*v*^ were lower than expected from muscle fiber data. Thus, in (89) at 25°C, *V*_*max*_ and *K*_*M*_^*v*^ were 5.3 *μ*m/s and 0.12 mM, respectively, whereas the corresponding values in (90) at 30°C were 7.2 *μ*m/s and 0.18 mM, respectively. It was previously suggested, based on modeling and experimental findings, that the higher *K*_*M*_^*v*^ value for muscle fibers and in vitro motility assays using HMM is due to nonlinear cross-bridge elasticity with buckling of S2. However, our modeling (Fig. 8) suggests that although both nonlinear cross-bridge elasticity and strain-independent rate function *k*_2_(*x*) are required to account for both the experimentally observed *V*_*max*_ and *K*_*M*_^*v*^ values, the change from strain dependence to strain independence of *k*_2_(*x*) is sufficient to account for high *K*_*M*_^*v*^. This finding, as well as the difference between thin filaments and pure actin filaments in the in vitro motility assay (89), points to greater complexity than previously assumed (20). Nevertheless, to conclude this section, the satisfactory predictions for both *V*_*max*_ and *K*_*M*_^*v*^ without strain dependence of *k*_2_(*x*), as a result of the introduction of nonlinear cross-bridge elasticity, resolves discrepancies between modeling and experimental findings (18).

### Physiological relevance

Possible evolutionary driving forces, i.e., physiological importance, behind the development of nonlinear cross-bridge elasticity were considered in the Results. First, we tested the idea that nonlinear cross-bridge elasticity might lead to higher thermodynamic efficiency compared to linear cross-bridge elasticity. Our analysis suggests that this would be the case if nonlinear elasticity is limited to the AM/AMD state. If the AMD_L_ state also exhibits nonlinear elasticity, the model predicts reduced efficiency because of increased ATP turnover rate that is not offset by increased power production. The increased ATP turnover rate in this case is due to increased cross-bridge attachment rate, something that does not occur in the model if the cross-bridge elasticity in the AMD_L_ state is linear. It should be mentioned here that our model is not optimized for analyzing the possibility of nonlinear elasticity in the AMD_L_ state. With linear elasticity in this state, attachment is effectively limited to a range of *x*-values between ∼4 and ∼12 nm (centered at *x* = *x*_1_ = 7.7 nm) as controlled by the elastic energy in the AMD_L_ state. This means that even if a myosin head may bind to more than one site along actin, such an effect is largely negligible, justifying the use of a single-site assumption (cf. (48)). However, the situation changes with nonlinear cross-bridge elasticity in which the lower stiffness at negative *x* would allow attachment to neighboring sites. Nevertheless, it is not clear to what extent the interactions with the thick filament backbone would allow a myosin molecule in the relaxed state to freely explore a wide range of *x*-values. In addition, it is likely that more than one mechanism limit the attachment range. For instance, a reduced overall attachment rate is expected with one additional state (with higher free-energy level than this AMD_L_ state) between cross-bridge attachment and P_i_-release, as suggested recently (47, 58) (see also (91)). Based on the above reasoning, we tentatively limited the possible attachment range. The removal of this limitation reduces sliding velocity and thermodynamic efficiency, whereas opposite effects are seen when the width of the allowed attachment range is reduced. We conclude that nonlinear elasticity in the AMD_L_ state may have important contractile effects. However, more complex models (21, 47, 92) are required to fully elucidate the issue.

Another effect of potential physiological importance, predicted by the model with nonlinear cross-bridge elasticity, is the increased number of attached cross-bridges during fast shortening compared to the case with linear cross-bridge elasticity. This effect is attributed to cross-bridges in their drag-stroke region. Because of their low stiffness, they do not reduce power output, nor do they increase muscle stiffness in proportion to the number of attached heads. However, importantly, if the load on the muscle is suddenly and unexpectedly increased, these negatively strained myosin heads constitute a reserve capacity to counteract excessive stretch. Thus, when these cross-bridges are positively strained during stretch, their stiffness will increase, resisting further elongation of the muscle. The version of the model that is associated with highest number of attached cross-bridges during fast shortening compared to the isometric case is that in which all states are assumed to have nonlinear cross-bridge elasticity (Figs. 6 *C* and 7 *B*). Interestingly, this is also the version that is in best agreement with the experimental data for ATP turnover rate (Fig. 9 *A*) and thermodynamic efficiency during shortening at different velocities. For instance, the experimental data for efficiency are dominated by maximal efficiency values close to 0.3 (68) (cf. Fig. 9 *B*). Therefore, one may speculate that the capacity to withstand sudden increases in load has been favored by natural selection at the expense of reduced maximal efficiency.

### Possible experimental tests to corroborate nonlinear cross-bridge elasticity in muscle

With regard to rigor conditions, two critical experiments are identified. First, it would be of interest to repeat the experiments of van der Heide et al. (24) in mammalian muscle fibers or myofibrils prepared in more conventional ways than by freeze-drying (24). Second, it is of critical importance (30, 33, 34, 38, 82) to settle the issue about the elastic properties (e.g., linear or nonlinear) of the myofilaments because these properties strongly affect mechanical experiments on muscle when tension is changing with time.

For active contraction, this study reports several testable predictions in addition to those we have already tested against existing experimental data. First, if the cross-bridge elasticity is nonlinear, the modeling suggests that the number of attached cross-bridges is higher during fast shortening than suggested by stiffness measurements. Additionally, the distribution of strains would be appreciably wider compared to isometric contraction. The latter effect is partly consistent with findings of Higuchi and Goldman in skinned muscle fibers, suggesting an extended drag-stroke region for the myosin head (93). A wide cross-bridge distribution, in terms of the variable *x* in our model, probably does not contradict evidence for a short working stroke from x-ray interference data (10) because such data would be expected to report the myosin head and lever arm conformations and not buckling of S2. However, this idea needs to be considered in greater detail.

Uncertainties as considered above regarding effects of nonlinear elasticity on the structure of the myosin head and lever arm, together with a wide *x*-distribution for attached cross-bridges, pose challenges for using x-ray diffraction and other structural methods to estimate the number of attached cross-bridges and their detailed distribution. Careful investigations are required before selecting alternative approaches to stiffness measurements for this purpose. An interesting possibility may be the use of fluorescence methods to probe the distance between fixed fluorophores (e.g., quantum dots) conjugated both to the myosin filament backbone and, e.g., to a light chain on the myosin lever arm. Possibly, such experiments may be performed in myofibrils or skinned muscle fibers if the preparation is placed on a surface for total internal reflection fluorescence microscopy to detect single molecules (94) with nanometer resolution (95).

Experimental tests would be more conclusive if it were possible to switch between conditions that produce linear cross-bridge elasticity (e.g., locking S2 to the thick filament backbone) and those that produce nonlinear elasticity (e.g., moving the myosin heads and S2 away from the thick filament backbone). For instance, if the cross-bridge elasticity in muscle is normally nonlinear, conversion to conditions with linear elasticity is predicted by our model to cause a number of changes. First, the stiffness in rigor would be appreciably increased (Fig. 3), and changes in stiffness upon length changes (Figs. 4 and 5) would no longer be observed. Second, in active contraction, the maximal velocity would be appreciably reduced, and the average stiffness would increase. Furthermore, altered relationships between the number of attached cross-bridges and stiffness (Fig. 6 *C*), between [MgATP] and velocity (Fig. 6 *D*), and between velocity and efficiency (Fig. 9 *B*) are predicted.

One condition that may be expected to reduce nonlinearity in cross-bridge elasticity, associated with S2 buckling, is osmotic compression of the muscle. By reducing the interfilament spacing, the myosin S2 region would be pushed toward the thick filament backbone, which might reduce tendencies for buckling. Although the effects of compression may be multifaceted (96, 97, 98), it is interesting to note that appreciably reduced shortening velocity is seen, together with increased stiffness both in rigor and during active contraction.

Ideally, the S2 domain should be more predictably cross-linked to, or uncoupled from, the thick filament backbone than is achievable by osmotic interventions. Alternatively, its buckling tendency should be reduced by increased persistence length (*L*_*P*_) or reduced contour length (*L*) because the critical force of buckling is proportional to the ratio *L*_*P*_/*L*^*2*^. It may be possible (although highly challenging) to achieve any of this using genetically engineered, transfected muscle cells (99). For instance, one may consider introduction of genetically modified S2 domains to allow chemical cross-linking, reducing the effective S2 length or increasing the S2 flexural rigidity (∝ *L*_*P*_). If myofibrils (99) can then be purified from these cells, mechanical investigations are possible. Alternatively, disease-causing mutations (100) might have already done the job, eliminating the need for cell cultivation and genetic engineering. However, whether the mutations actually change the elastic properties must be independently investigated, e.g., in single-molecule studies similar to those in (29).

## Conclusions

Bottom-up-defined cross-bridge models for rigor and active contraction give faithful reproductions of a wide range of experimentally observed results on the assumption that the cross-bridge elasticity has nonlinear characteristics similar to those in single-molecule studies (29). A somewhat surprising finding was that the substantial nonlinearity in single molecules does not reproduce the full degree of the nonlinearity of the force-extension relation of rigor fibers. Therefore, although our analysis of the rigor condition is in agreement with cross-bridge nonlinearity of similar type in cells as in single molecules, the results also indicate nonlinear myofilament elasticity. Also, the analysis of active contraction supports nonlinear cross-bridge elasticity in muscle cells. Some central experimental findings during active contraction are well accounted for if only the AM/AMD state exhibits nonlinear elasticity. Although our results are also consistent with nonlinear cross-bridge elasticity in other states, more complex models will be required to fully evaluate this issue. Finally, we consider physiological implications of the results and suggest experimental studies that may be used to test model predictions. Such studies are of utmost importance because our results challenge a long-standing paradigm (4, 22, 30, 101) of linear elasticity in cross-bridges and myofilaments in muscle.

## Author Contributions

A.M. and D.E.R. initiated and conceived the study. A.M. developed the model, ran model simulations, and analyzed model data in relation to experiments. M.P. and N.S. analyzed key data. A.M. coordinated the project, but all authors contributed to data interpretation and writing of the manuscript as well as final approval of the manuscript.

## Supporting Material

Document S1. Supporting Materials and Methods, Figs. S1 and S2, and Tables S1 and S2

Document S2. Article plus Supporting Material
