# Supplementary material for: Nonlinear Actomyosin Elasticity in Muscle?
Source: Biophys J. 2018 Dec 13;116(2):330–46. doi: 10.1016/j.bpj.2018.12.004 (PMC6350078; doi:10.1016/j.bpj.2018.12.004)
Supplement: Document S1. Supporting Materials and Methods, Figs. S1 and S2, and Tables S1 and S2 [file mmc1.pdf]

Biophysical Journal, Volume *116*

Supplemental Information

Nonlinear Actomyosin Elasticity in Muscle?

Alf Månsson, Malin Persson, Nabil Shalabi, and Dilson E. Rassier

Supplementary Methods

Simulations of active contraction with "periodic boundary conditions"

It was important to explicitly consider the fact that a cross-bridge attaching at one actin site may be available for attachment at a range of x-values at a neighbouring actin site 36 nm towards the barbed end of the actin filament. This is related to the wide range for integration in Eqs. 22-25 in the main paper. As the same conditions are expected to apply to all sites, the phenomenon may be treated formally by adding one further AM/AMD state (AM^{36}) with its free energy minimum (2 k_BT) at x=36 nm. We denote this approach, "integration with periodic boundary conditions". Simulations in the range of x-values starting at 14 nm and ending at x=-22 nm (i.e. over 36 nm) were first iterated with the aim to set the initial values at x=14 nm (right dotted vertical line in Fig. S2) for the MT, MDP and AM^{36} states equal to the values of corresponding states at x=-22 nm. Upon completion of this iteration process, the initial values for the MT and MDP states at x=14 nm were equal to the calculated values at x=-22 nm and the initial value for the AM^{36} state was equal to the AM/AMD value at x=-22 nm to a fractional accuracy of < 0.001. Then, the simulation of the state probability distributions and the subsequent integrations were repeated to obtain force, stiffness, ATPase and Na (cf. Eqs. 22-25). For the calculations with periodic boundary conditions, we used rate functions and force-functions consistent with the free energy diagrams in Fig. S2B where the orange dashed curve with minimum at x=36 nm is associated with the AM^{36} state. The cross-bridge distribution showing the population of the different states (including the AM^{36} state) for the case of shortening with non-linear cross-bridge elasticity at 14000 nm/s is depicted in Fig. S2A. This distribution corresponds to that without periodic boundary conditions (i.e. integration from 14 to -70 nm) in Fig. 7A in the main paper. The latter method is generally used below but critical control simulations using integration with periodic boundary conditions are reported throughout the paper.

Supplementary Figures

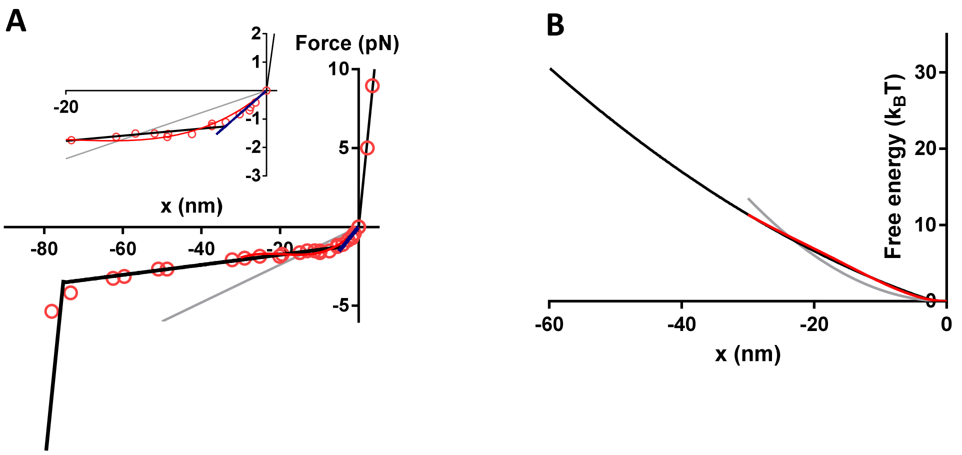

Fig. S1. Force and free energy vs cross-bridge strain in model simulations and single molecule experiments. A. Force-extension relationships showing force vs cross-bridge strain (x)**.** Red circles correspond to measurements from Fig. 1 of Kaya and Higuchi (1). The full red line corresponds to third order polynomial fit to the experimental data ($F_{KH}(x - 5.5i)$= 0.296x + 0.0161x^2 + 0.00028x^3) for x<0 nm and a linear fit for x>0 nm. This representation (x>-25 nm) was used in simulation of rigor muscle properties. Black full lines: Piecewise linear approximation of the experimental data. The latter representation (x>-85 nm) was used to describe the elasticity of the AM/AMD state for analysis of actively contracting muscle. Grey line represents linear fit to the experimental data in the range x∈[-25,0] nm. The line (slope: 0.12 pN/nm) was forced through the origin. This representation was used to describe the elastic properties of the AMD_L and the AMD_H states after shifting the zero force level along the x-axis to x_1 and x_2, respectively (cf. main Fig. 2). **B**. Free energy attributed to cross-bridge elasticity in the AM/AMD state for x<0 nm, estimated by integration of the different representations of the force-extension curve in Fig. S1A. Coding in color and grey scale as in A.

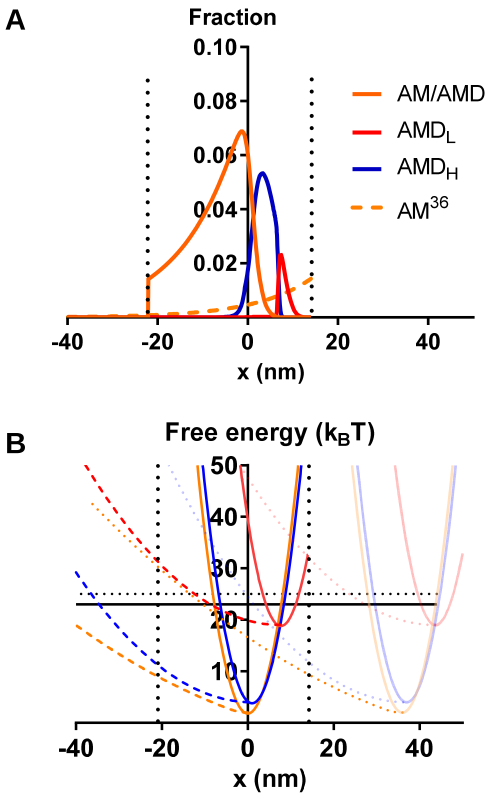

Fig. S2. Control simulations using periodic boundary conditions as described in text. A. Population of different cross-bridge states during shortening near maximum velocity (14000 nm/s) in version of model with non-linear cross-bridge elasticity and numerical integration with periodic boundary conditions (see text). The integration is limited to the range [-22, 14] nm (between dotted vertical lines). The population of each state at a given value of x is shown as the fraction of the total number of heads at that x-value. Note that effectively, the AM/AMD distribution (full orange line) is displaced by 36 nm to the right (dashed orange line) corresponding to the state AM^{36} with its minimum free energy at x=36 nm (see Fig. S2B). This is done after iteratively ensuring that the initial values correspond to relevant values at x=-22 nm (see text). The corresponding distribution without periodic boundary conditions is illustrated in Fig. 7B in the main paper. **B.** Free energy diagrams for cross-bridge states at two neighbouring sites along an actin filament. The site with the AM/AMD state centred at zero corresponds directly to the free energy diagrams in Fig. 2B in the main paper. For the site at 36 nm, we only consider the AM/ADP state, here denoted as the AM^{36} state (dotted orange line). The vertical dotted lines indicate the boundaries for numerical integration.

Supplementary Tables

Table S1. Parameter values[a] determining shape of free energy diagrams for simulating active contraction of fast mammalian muscle at 30 ^{o}C

**Parameter**	**Explanation**	**Numerical value linear**	**Numerical value non-linear**[b]	**Literature values, references**
x_w	x-position of free energy minimum in AMDP state	7.7 nm		Equal to x_1, (2) and references therein
x_1	x-position of free energy minimum in AMD_L state	7.7 nm		7-9 nm, (2) and references therein
x_2	x-position of free energy minimum in AMD_H state	1.0 nm		1-2 nm, (2) and references therein
x_3	x-position of free energy minimum in AM/AMD state	0 nm		See text
ΔG_w	Difference in free energy between MDP and AMDP state	2.5 k_BT		~0-2.5 k_BT, (3, 4)
$\Delta G_{AMDP\text{-}AMDL}$	Difference in minimum free energy between AMDP and AMD_L state	0.7 k_BT $\ln([P_i]/K_C)$ + elastic		See K_C below; (5, 6)
$\Delta G_{AMDL\text{-}AMDH}$	Difference in minimum free energy between AMD_L and AMD_H state	15 k_BT		11-19 k_BT; (2) and references therein
$\Delta G_{AMDH\text{-}AMD}$	Difference in minimum free energy between AMD_H and AMD state	2 k_BT		1-2 k_BT, (2) and references therein
ΔG_{ATP}	Free energy of ATP turnover	13.1 + ln ([MgATP]/([MgADP][Pi]) k_BT		Free energy of ATP-hydrolysis, (7)
ks	Cross-bridge stiffness, strongly bound states	2.5 (rigor)-2.8 (active) pN/nm	0.03 -2.8 pN/nm (Eqs. 9a-11e)	(2) and references therein
ksw	Cross-bridge stiffness, weakly bound states	0.0001 pN/nm[c]	-	See text

[a] The parameter values were from steady-state and non-steady state analyses applied to two-headed myosin motor fragments from fast skeletal muscle of rabbit at 30°C, ionic strength 130-200 mM, pH 7-8 unless otherwise stated.

[b]Only given if different from the linear case

Table S2. Parameter values[a] defining rate functions and kinetic constants for simulating active contraction of fast mammalian muscle at 30 ^{o}C.

Para-meter	Explanation	Numerical value, linear	Numerical value, non-linear[b]	Litterature range, references
$k_{+3} + k_{-3}$	Recovery stroke, ATP hydrolysis rate constants	220 s^{-1}		200-500 $s^{-1,}$ (13, 14) and references therein Assuming Q_{10} in range 3-4 (13)[c].
K_3	Equilibrium constant for recovery stroke and ATP hydrolysis	10		From myosin subfragment 1 from fast muscle, (13, 14) and references therein
$K_{LH}(x)$	Equilibrium constant for rapid force generation	cf. Eqs. 15-17		Follows from free energy curves defined by parameter values above
$k_5(x_2)$	Maximum rate of strain dependent transition before ADP relase	2000 s^{-1}		Fit to force-velocity relationship (4, 9, 15)
Kc	Phosphate dissociation constant	10 mM		~1-10 mM From fast skinned muscle fiber phosphate transients, from data at 20-25 ^{o}C, (5)
k_{b0}	Rate limiting step for phosphate release and cross-bridge attachment into strongly bound actomyosin states	100 s^{-1}		~100 s^{-1}, From fast skinned muscle fiber phosphate transients, from data at 20-25 ^{o}C[b], (5)
x_{crit}	Bell-type strain-dependence of ATP induced detachment rate ($k_2(x)$)	0.6 nm	0 nm	< 0.2 nm, From myosin subfragment 1 of fast mouse muscle at 20 ^{o}C and ionic strength < 50 mM, (16)
k_6	Rate constant of ADP dissociation from AMD-state	5000 s^{-1}		>3500 s^{-1}, Fast rabbit myosin subfragment 1, (17)
Physiological [Pi]		0.5 mM		~ 0.5 mM, (18)
K_1	ATP association constant to AM state	1.7 mM^{-1}		1.7 mM^{-1}, From fast rabbit myosin subfragment 1, (17)
$k_2(0)$	ATP induced dissociation rate constant at x=0 nm	1800 s^{-1}	1600 s^{-1}	1600 - 2000 s^{-1}, fast rabbit myosin subfragment 1; Temperature corrected (Q_{10}=2.3) from 25 ^{o}C (17).

[a] The parameter values were from steady-state and non-steady state analyses of two-headed myosin fragments from fast skeletal muscle of rabbit at 30°C, ionic strength 130-200 mM, pH 7-8 unless otherwise stated.

[b]Only given if different from the linear case

[c]The parameters k_3 and, particularly k_{b0}, are key determinants of the maximum actin activated ATPase and the rate of rise of isometric force (14). Some variability is seen in the literature both at a given temperature and in the temperature dependence (6, 12, 14, 19). However, the values used here account well, in terms of the present model, for the maximum actin activated ATPase (6) at 30 ^{o}C as well as the rate of rise of isometric force (14) extrapolated to 30 ^{o}C (4).

Supplementary References

1. Kaya, M., and H. Higuchi. 2010. Nonlinear elasticity and an 8-nm working stroke of single myosin molecules in myofilaments. Science 329:686-689.
2. Mansson, A., M. Usaj, L. Moretto, and D. E. Rassier. 2018. Do Actomyosin Single-Molecule Mechanics Data Predict Mechanics of Contracting Muscle? Int. J. Mol. Sci. 19.
3. Brenner, B., L. C. Yu, L. E. Greene, E. Eisenberg, and M. Schoenberg. 1986. Ca2+-sensitive cross-bridge dissociation in the presence of magnesium pyrophosphate in skinned rabbit psoas fibers. Biophys. J. 50:1101-1108.
4. Mansson, A. 2016. Actomyosin based contraction: one mechanokinetic model from single molecules to muscle? J. Muscle Res. Cell Motil. 37:181-194.
5. Dantzig, J. A., Y. E. Goldman, N. C. Millar, J. Lacktis, and E. Homsher. 1992. Reversal of the cross-bridge force-generating transition by photogeneration of phosphate in rabbit psoas muscle fibres. J Physiol 451:247-278.
6. Brenner, B., and E. Eisenberg. 1986. Rate of force generation in muscle: correlation with actomyosin ATPase activity in solution. Proc. Natl. Acad. Sci. U. S. A. 83:3542-3546.
7. Pate, E., and R. Cooke. 1989. A model of crossbridge action: the effects of ATP, ADP and Pi. J. Muscle Res. Cell Motil. 10:181-196.
8. Mansson, A. 2010. Actomyosin-ADP states, inter-head cooperativity and the force-velocity relation of skeletal muscle. Biophys. J. 98:1237-1246.
9. Albet-Torres, N., M. J. Bloemink, T. Barman, R. Candau, K. Frölander, M. A. Geeves, K. Golker, C. Herrmann, C. Lionne, C. Piperio, S. Schmitz, C. Veigel, and A. Månsson. 2009. Drug effect unveils inter-head cooperativity and strain-dependent ADP release in fast skeletal actomyosin. J. Biol. Chem. 284:22926–22937.
10. Veigel, C., J. E. Molloy, S. Schmitz, and J. Kendrick-Jones. 2003. Load-dependent kinetics of force production by smooth muscle myosin measured with optical tweezers. Nature Cell Biol. 5:980-986.
11. Whittaker, M., E. M. Wilson-Kubalek, J. E. Smith, L. Faust, R. A. Milligan, and H. L. Sweeney. 1995. A 35-A movement of smooth muscle myosin on ADP release. Nature 378:748-751.
12. Rahman, M. A., M. Usaj, D. E. Rassier, and A. Mansson. 2018. Blebbistatin Effects Expose Hidden Secrets in the Force-Generating Cycle of Actin and Myosin. Biophys. J. 115:386-397.
13. Woledge, R. C., N. A. Curtin, and E. Homsher. 1985. Energetic aspects of muscle contraction. Academic Press, London.
14. Sleep, J., M. Irving, and K. Burton. 2005. The ATP hydrolysis and phosphate release steps control the time course of force development in rabbit skeletal muscle. J. Physiol. 563:671-687.
15. Persson, M., E. Bengtsson, L. ten Siethoff, and A. Mansson. 2013. Nonlinear cross-bridge elasticity and post-power-stroke events in fast skeletal muscle actomyosin. Biophys. J. 105:1871-1881.
16. Capitanio, M., M. Canepari, M. Maffei, D. Beneventi, C. Monico, F. Vanzi, R. Bottinelli, and F. S. Pavone. 2012. Ultrafast force-clamp spectroscopy of single molecules reveals load dependence of myosin working stroke. Nature Meth. 9:1013-1019.
17. Nyitrai, M., R. Rossi, N. Adamek, M. A. Pellegrino, R. Bottinelli, and M. A. Geeves. 2006. What limits the velocity of fast-skeletal muscle contraction in mammals? J. Mol. Biol. 355:432-442.
18. Debold, E. P., M. A. Turner, J. C. Stout, and S. Walcott. 2011. Phosphate enhances myosin-powered actin filament velocity under acidic conditions in a motility assay. Am. J. Physiol. Regul. Integr. Comp. Physiol. 300:R1401-1408.
19. Zhao, Y., and M. Kawai. 1994. Kinetic and thermodynamic studies of the cross-bridge cycle in rabbit psoas muscle fibers. Biophys. J. 67:1655-1668.
